# The acetylation of cyclin-dependent kinase 5 at lysine 33 regulates kinase activity and neurite length in hippocampal neurons

**DOI:** 10.1038/s41598-018-31785-9

**Published:** 2018-09-12

**Authors:** Juhyung Lee, Yeon Uk Ko, Yuhyun Chung, Nuri Yun, Myungjin Kim, Kyungjin Kim, Young J. Oh

**Affiliations:** 1 Department of Systems Biology, Yonsei University College of Life Science and Biotechnology, Seoul, 03722 Republic of Korea; 2 Laboratory of Molecular Biology, National Institute of Diabetes and Digestive and Kidney Diseases, National Institutes of Health, Bethesda, MD 20892 USA; 3 Laboratory of Neurobiochemistry, Department of Neural Development and Disease, Korea Brain Research Institute, Daegu, 41068 Republic of Korea; 4 Department of Brain and Cognitive Sciences, Daegu Gyeongbuk Institute of Science and Technology (DGIST), Daegu, 42988 Republic of Korea

## Abstract

Cyclin-dependent kinase 5 (CDK5) plays a pivotal role in neural development and neurodegeneration. CDK5 activity can be regulated by posttranslational modifications, including phosphorylation and S-nitrosylation. In this study, we demonstrate a novel mechanism by which the acetylation of CDK5 at K33 (Ac-CDK5) results in the loss of ATP binding and impaired kinase activity. We identify GCN5 and SIRT1 as critical factor controlling Ac-CDK5 levels. Ac-CDK5 achieved its lowest levels in rat fetal brains but was dramatically increased during postnatal periods. Intriguingly, nuclear Ac-CDK5 levels negatively correlated with neurite length in embryonic hippocampal neurons. Either treatment with the SIRT1 activator SRT1720 or overexpression of SIRT1 leads to increases in neurite length, whereas SIRT1 inhibitor EX527 or ectopic expression of acetyl-mimetic (K33Q) CDK5 induced the opposite effect. Furthermore, the expression of nuclear-targeted CDK5 K33Q abolished the SRT1720-induced neurite outgrowth, showing that SIRT1 positively regulates neurite outgrowth via deacetylation of nuclear CDK5. The CDK5 activity-dependent increase of neurite length was mediated by enhanced transcriptional regulation of BDNF via unknown mechanism(s). Our findings identify a novel mechanism by which acetylation-mediated regulation of nuclear CDK5 activity plays a critical role in determining neurite length in embryonic neurons.

## Introduction

Cyclin-dependent kinase 5 (CDK5), a proline-directed serine/threonine kinase, is closely related to other cyclin-dependent kinases and is expressed in various tissues, although its highest level is found in the brain^1–3^. However, CDK5 kinase activity is detected only in postmitotic neurons via its association with the neuron-specific activators p35 or p39^4,5^. In contrast to other CDKs that promote cell proliferation, CDK5 plays a role in regulating proper brain development, neuronal maturation and neuronal death^6^. For example, CDK5 is hyperactivated by the conversion of p35 to p25 by the calcium-dependent protease calpain under pathological conditions^7,8^. Dysregulation of CDK5 kinase activity is linked to an array of neurodegenerative diseases^9^. During early brain development, CDK5 expression and kinase activity closely correlate with the extent of neuronal differentiation^2,10,11^. More specifically, CDK5 is involved in axonal and dendritic growth, neuronal migration and synapse development via the phosphorylation of specific substrates in different cellular compartments^12–15^. Consequently, CDK5 knock-out (KO) or p35/p39 double-KO mice exhibit perinatal lethality characterized by cortical layering, lamination and fasciculation failure^16,17^.

In addition to activation by virtue of association with p35 (or its truncated form p25) and p39, post-translational modification of CDK5 itself is an additional determinant that influences kinase activity. Although CDK5 is not needed to be phosphorylated to become active, it has been reported that phosphorylation at T14 in a glycine-rich loop (G loop; residues 11–16) of CDK5 by one or more unknown kinase down-regulates kinase activity *in vitro*^18^. In contrast, phosphorylation of the adjacent Y15 by c-Abl, Fyn and EPHA4 leads to increased kinase activity^19–21^, although a contradicting report is available^22^. Interestingly, the c-Abl-induced phosphorylation of CDK5 is enhanced by Cables, a CDK5-interacting protein in the brain, and promotes neurite outgrowth in cultured cortical neurons^21^. Furthermore, Fyn-mediated phosphorylation of CDK5 in response to Sema3A signaling results in growth-cone collapse, suggesting that Y15 phosphorylation is potentially involved in axon and dendrite guidance^20^. Similarly, S-nitrosylation also plays an important role in regulating CDK5 activity^23,24^. C83 and C157 of CDK5 are S-nitrosylated and these modifications lead to kinase activity inhibition^24^. Consequently, mutant CDK5 lacking these cysteine residues delays dendritic development in hippocampal neurons. In contrast, another group reported an opposing outcome for the S-nitrosylation of CDK5 in Aβ_1–42_ and NMDA-induced dendritic spine loss^23^.

While searching for additional posttranslational modifications of CDK5, we previously found that general control non-derepressible 5 (GCN5, KAT2A) acetylates CDK5 at K33^25^. Prompted by this observation, we specifically investigated (i) whether CDK5 acetylation affects its kinase activity and (ii) whether it is involved in neuronal differentiation. Here, we found that acetylation of CDK5 at K33 inhibited its kinase activity via the loss of ATP binding. We further demonstrated that (de)acetylated CDK5 levels and concomitant kinase activity were controlled by SIRT1 in developing hippocampal neurons. Based on both pharmacological and genetic evidence, acetylated CDK5 levels inversely correlated with the extent of total neurite length and the length of the longest neurite. Transcriptional regulation of BDNF involved CDK5 kinase activity-dependent neurite extension via one or more unknown mechanism independent of MeCP2 and CREB phosphorylation. Collectively, our findings suggest a novel mechanism to regulate CDK5 activity in the nucleus and highlight the crucial role of CDK5 in determining neurite length in the developing brain.

## Results

### Acetylated CDK5 loses its kinase activity via impaired binding to ATP

**Figure 1:**
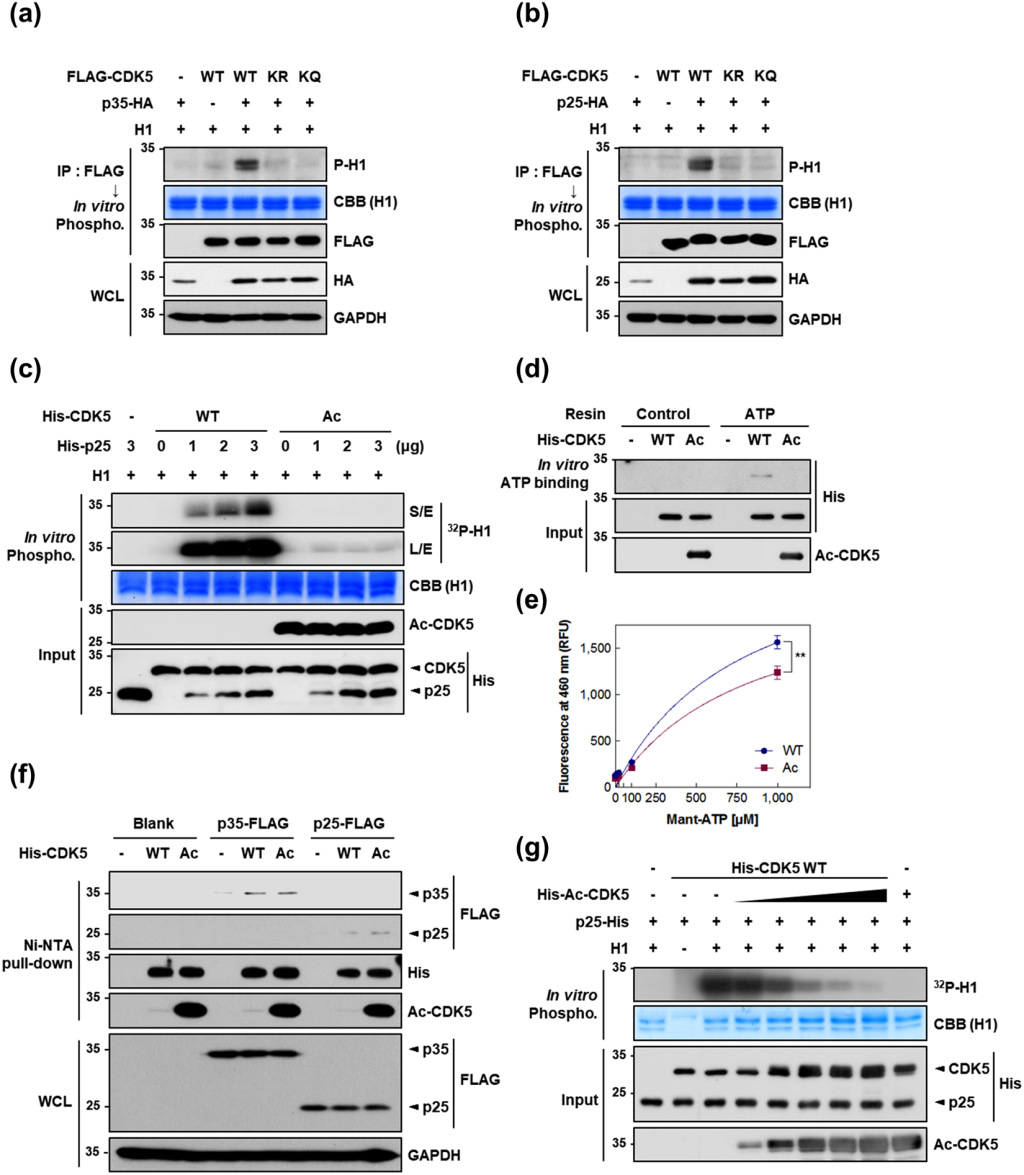
Acetylation of CDK5 at K33 causes a loss of kinase activity due to impaired ATP binding. (**a**,**b**) HEK293 cells were transfected with either FLAG-tagged wild type mouse CDK5 (WT), an acetyl-null mutant (K33R; KR) or a mimetic mutant (K33Q; KQ) of CDK5 in the presence of (**a**) p35-HA or (**b**) p25-HA. Lysates were immunoprecipitated (IPed) with an anti-FLAG antibody and then subjected to an *in vitro* phosphorylation assay using histone H1 as a substrate. The resulting phosphorylated H1 (P-H1) was visualized via immunoblot analysis (IB) with an anti-phospho-H1 antibody. Coomassie brilliant blue (CBB) staining for H1 was used as a loading control. Immunoprecipitates or whole cell lysates (WCLs) were resolved by SDS-PAGE and subjected to IB with the indicated antibodies. Anti-glyceraldehyde-3-phosphate dehydrogenase (GAPDH) was used as a loading control for WCL. (**c**) Bacterially purified, recombinant His-tagged CDK5 WT or K33-acetylated CDK5 (Ac-CDK5; Ac) was subjected to an *in vitro* phosphorylation assay in the presence of H1, [γ-^32^P]ATP and the indicated doses of recombinant p25. The resulting phosphorylated H1 was visualized by autoradiography. Inputs were resolved by SDS-PAGE and subjected to IB with the indicated antibodies. (**d**) Recombinant His-CDK5 WT or His-Ac-CDK5 was incubated with or without resin conjugated to ATP. After washing, the resulting ATP-bound CDK5 was resolved by SDS-PAGE and visualized by IB with an anti-His antibody. Input signals were measured by IB with the indicated antibodies. (**e**) Recombinant His-CDK5 WT (blue-filled circles) or His-Ac-CDK5 (magenta-filled rectangles) was titrated with increasing concentration of mant-ATP. Nonlinear regression was performed to obtain a best-fit curve for a specific binding [Y = Bmax*X/(Kd + X)] and the summary of binding parameters were shown in Supplementary Table 1. X-axis represents the varying concentration of mant-ATP as indicated. Y-axis represents the relative fluorescence intensity of specific binding, where Bmax is maximum specific binding and Kd is equilibrium binding constant. Wilcoxon matched-pairs rank test was employed to test the binding difference between CDK5 WT and Ac-CDK5 (^**^P = 0.004; Spearman correlation coefficient, rs = 0.976; n = 3). (**f**) Lysates from HEK293 cells expressing p35-FLAG or p25-FLAG were incubated with recombinant His-CDK5 WT or His-Ac-CDK5 bound to Ni-NTA beads. Reaction mixtures were subjected to pull-down and subsequent IB with the indicated antibodies. An anti-FLAG antibody was employed to visualize the extent of CDK5-bound p35 or p25. WCLs were subjected to IB with the indicated antibodies. (**g**) Recombinant His-CDK5 WT plus increasing amounts of recombinant His-Ac-CDK5 was subjected to an *in vitro* phosphorylation assay in the presence of H1 and [γ-^32^P]ATP. Phosphorylated H1 levels were visualized by autoradiography.

Employing mass spectral analysis and immunological assays, we demonstrated acetylation at K33 of CDK5 in HEK293 cells by GCN5, a member of the GCN5-related N-acetyltransferase (GNAT) family^25^. The K33 residue in CDK5 is highly conserved among various species and is necessary for ATP binding^25^. We thus speculated that the acetylation of CDK5 at K33 might alter its kinase activity. To address this question, HEK293 cells were transfected with CDK5 wild type (WT) or CDK5 mutants in which K33 was substituted with arginine (K33R; acetyl null) or glutamine (K33Q; acetyl mimetic) in combination with p35 or p25. The kinase activities of the immunoprecipitates were assessed using histone H1 (H1) protein as a CDK5 substrate. The *in vitro* kinase assays showed that immunoprecipitated CDK5 WT significantly phosphorylated H1 in the presence of p35 or p25 (Fig. 1a,b). In contrast, neither of the two mutants maintained their kinase activity regardless of the presence or absence of CDK5 activators. Based on previous studies^2^, we reasoned that K33 mutation in CDK5 would lead to a loss of kinase activity. In support of this notion, CDK5 K33T has often been employed as a dominant-negative form of CDK5^8^. To investigate the consequences of acetylation of CDK5 at K33 (described below as Ac-CDK5), CDK5 WT and Ac-CDK5 proteins were purified for use in *in vitro* kinase assays utilizing a protocol to perform site-directed acetylation in a bacterial system (Supplementary Fig. 1a)^26^. To specifically detect the Ac-CDK5 protein, we generated a polyclonal antibody by immunizing rabbits with a short synthetic peptide sequence spanning acetylated K33 in CDK5. In immunoprecipitation and immunoblot analyses, this antibody detected only a single band in the immunoprecipitates obtained from HEK293 cells transfected with GCN5 and CDK5 WT (Supplementary Fig. 2). No band was detected in HEK293 cells transfected with CDK5 K33R or K33Q. We further confirmed that this antibody specifically recognizes bacterially purified Ac-CDK5 (Supplementary Fig. 1b). To our surprise, the kinase activity of the purified Ac-CDK5 was abolished even in the presence of p25, whereas the kinase activity of non-acetylated CDK5 WT was enhanced with increasing doses of p25 (Fig. 1c). Among the many possible explanations for this result, we notably found that Ac-CDK5 could not bind to ATP resin (Fig. 1d). As we hypothesized that the acetylation of CDK5 may abolish ATP binding, we utilized the fluorescent ATP analogue mant-ATP (2′/3′-O-(N-methylanthraniloyl)-adenosine-5′-triphosphate) to directly assess the mant-ATP-CDK5 interactions. When incubated with varying concentrations of mant-ATP, CDK5 WT showed much greater fluorescence intensity than Ac-CDK5 (Fig. 1e). We found that CDK5 WT showed higher Bmax and binding potential as compared to those of Ac-CDK5 (Supplementary Table 1). It is of note that this elevated binding affinity of WT to mant-ATP was not observed in the presence of excess ATP (Supplementary Fig. 3a). In contrast, there was no binding difference between CDK5 WT and Ac-CDK5 against mant-ADP (Supplementary Fig. 3b). Next, we assessed the involvement of an altered interaction between Ac-CDK5 and p35 or p25. Cellular lysates from p35- or p25-transfected HEK293 cells were incubated with purified CDK5 WT or Ac-CDK5 on Ni-NTA agarose beads. Ac-CDK5 exhibited no detectable changes in its binding to any of the activators compared with CDK5 WT binding (Fig. 1f). We then examined whether CDK5 acetylation leads to an altered ability to recognize substrate. Increasing amount of Ac-CDK5 efficiently hindered the phosphorylating activity from the fixed amount of CDK5 WT proteins *in vitro* (Fig. 1g), suggesting that substrate recognition is not affected by the acetylation of CDK5 at K33. Taken together, our data indicated that the observed loss of Ac-CDK5 kinase activity is primarily caused by impaired ATP binding affinity.

### GCN5-mediated K33-acetylation leads to a loss of CDK5 nuclear kinase activity

**Figure 2:**
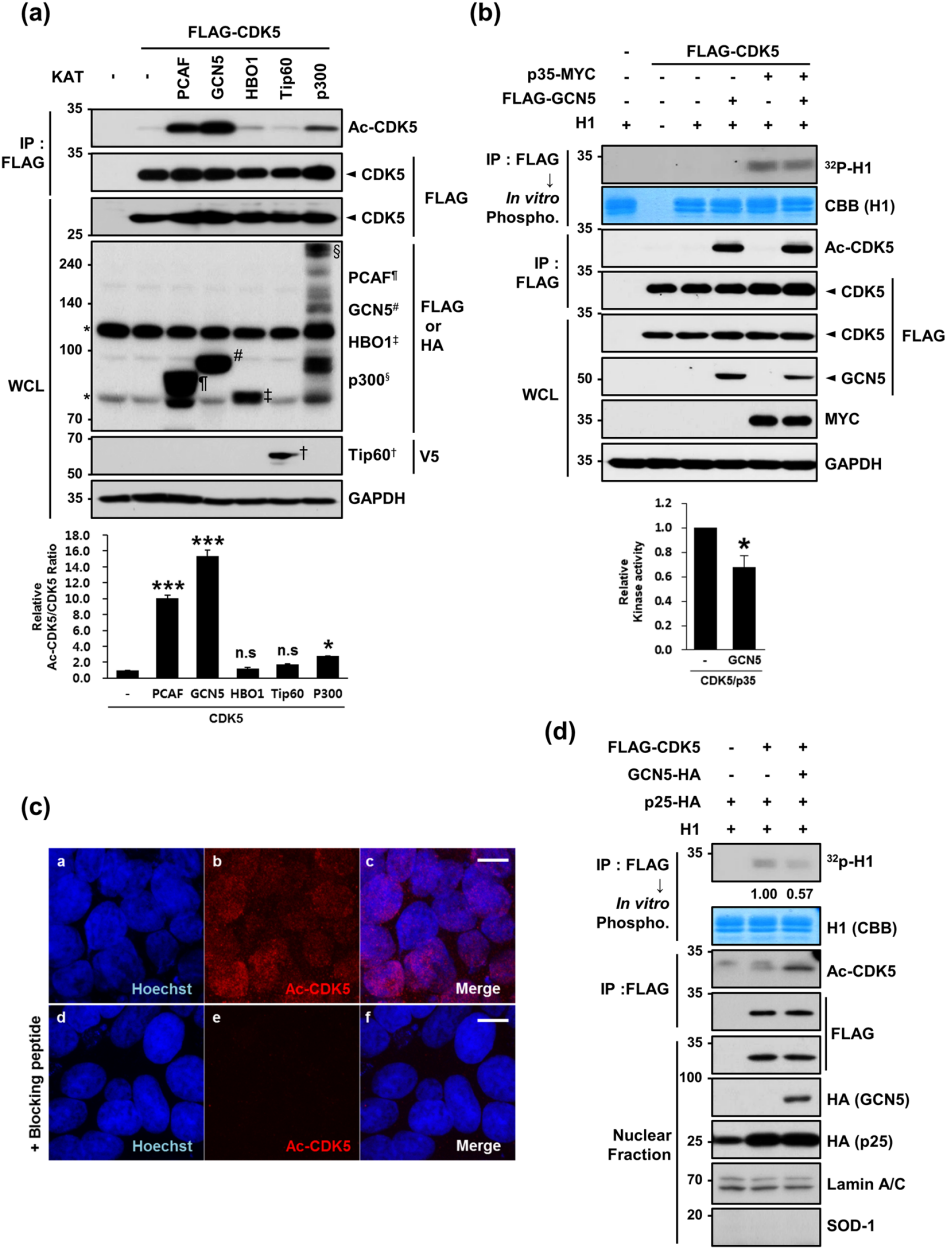
GCN5 acetylates CDK5 at K33 in the nucleus. (**a**) Lysates obtained from HEK293 cells expressing FLAG-CDK5 plus one of the indicated KAT vectors were subjected to IP with an anti-FLAG antibody followed by IB with an anti-Ac-CDK5 antibody or anti-FLAG antibody. The intensity of the Ac-CDK5 band was measured using Image-J software and normalized to FLAG-CDK5. The fold change over the control (value = 1) is indicated at the bottom of the blot. WCLs were subjected to IB analysis with the indicated antibodies. Each KAT band is marked by the indicated letters. The asterisk indicates non-specific bands. After normalization to FLAG-CDK5, the fold intensity of Ac-CDK5 versus the control (value = 1) was indicated. The bar represents the mean ± S.D from three independent experiments. ^***^p < 0.001; ^*^p < 0.05; n.s, not significant. (**b**) Lysates harvested from HEK293 cells expressing FLAG-CDK5 alone or in combination with p35-MYC and/or FLAG-GCN5 were subjected to IP with an anti-FLAG antibody. The bound CDK5 was incubated in the presence of H1 and [γ-^32^P]ATP and visualized by autoradiography. The relative kinase activity of CDK5/p35 was expressed as the fold change over the control (value = 1). The bar represents the mean ± S.D from 3 independent experiments. ^*^p < 0.05. (**c**) HEK293 cells were immunostained with an anti-Ac-CDK5 antibody. Staining specificity was confirmed by pre-incubating with the blocking peptide (EIVAL(acK)RVRLD) that was used to raise the antibody. The nuclei were counterstained with Hoechst dye. Confocal microscopy images are shown. The scale bar represents 10 μm. (**d**) HEK293 cells transfected with the indicated combinations of constructs were subjected to cellular fractionation. The resulting nuclear fractions were IPed with an anti-FLAG antibody and subsequently subjected to either IB with an anti-Ac-CDK5 or anti-FLAG antibody or an *in vitro* phosphorylation assay in the presence of H1 and [γ-^32^P]ATP. Signals from the phosphorylated H1 were visualized by autoradiography. The fold change over the control (value = 1) is indicated. Nuclear fractions were subjected to IB with the indicated antibodies. Anti-SOD-1 and anti-lamin A/C antibodies were employed to verify the purity of the nuclear fractions.

To specifically examine which lysine acetyltransferases (KATs) are responsible for CDK5 acetylation at K33, we transfected HEK293 cells with CDK5 plus one of the KATs (PCAF, GCN5, HBO1, Tip60 and p300) and measured K33-acetylation levels using an anti-Ac-CDK5 antibody. Although the expression levels of several KATs varied in HEK293 cells, PCAF, GCN5 and p300 KATs effectively acetylated K33 of CDK5, and GCN5 demonstrated the highest efficacy (Fig. 2a). Although the possibility of involvement of five transferases in acetylation of CDK5 was not determined *in vivo*, we considered highest efficacy of acetylation by GCN5 and a predominant expression of GCN5 over PCAF in the brain^27,28^. Therefore, we focused to test whether the expression of GCN5 altered CDK5 kinase activity. Co-expression of GCN5 plus p35 in HEK293 cells decreased CDK5 kinase activity of CDK5 by 20% (Fig. 2b). Interestingly, immunofluorescence analysis with an anti-Ac-CDK5 antibody revealed that Ac-CDK5 was primarily localized in the nucleus (Fig. 2c,a–c), and this staining was blocked by pre-incubation with a short Ac-CDK5 peptide (Fig. 2c,d–f). Unexpectedly, we found that Ac-CDK5 assembled into discrete nuclear foci in HEK293 cells overexpressing GFP-tagged GCN5 (Supplementary Fig. 4a,a–f). In contrast, a GCN5 catalytic mutant (E575Q) did not cause any discernible formation of nuclear foci, suggesting that this event is dependent on the catalytic activity of GCN5 (Supplementary Fig. 4a,g–i). Again, these foci were not detected when the antibody was blocked by pre-incubation with a short Ac-CDK5 peptide (Supplementary Fig. 4a,j–l). When HEK293 cells transfected with GCN5 were co-stained with several subnuclear domain markers, Ac-CDK5 foci co-localized with a spliceosome marker (SC-35; Supplementary Fig. 4b,a–c) but not with a nucleoli marker (C23; Supplementary Fig. 4b,d–f) or with γ-H2AX foci regardless of camptothecin treatment (Supplementary Fig. 4b,g–l)^29^. Because the basal nuclear staining pattern of Ac-CDK5 in HEK293 cells did not exhibit any discernible foci, we speculated that GCN5-mediated generation of nuclear Ac-CDK5 above a certain threshold level is capable of inducing focal formation at the spliceosome. Although we did not attempt to determine the significance of this event, we performed an *in vitro* kinase assay to observe whether GCN5-mediated formation of Ac-CDK5 foci is linked to a decrease in CDK5 kinase activity in the nucleus. Based on immunoblot analysis with the nuclear fraction, the GCN5-mediated increase in Ac-CDK5 was linked to reduced CDK5 activity (Fig. 2d).

### SIRT1 and SIRT2 effectively deacetylate Ac-CDK5

**Figure 3:**
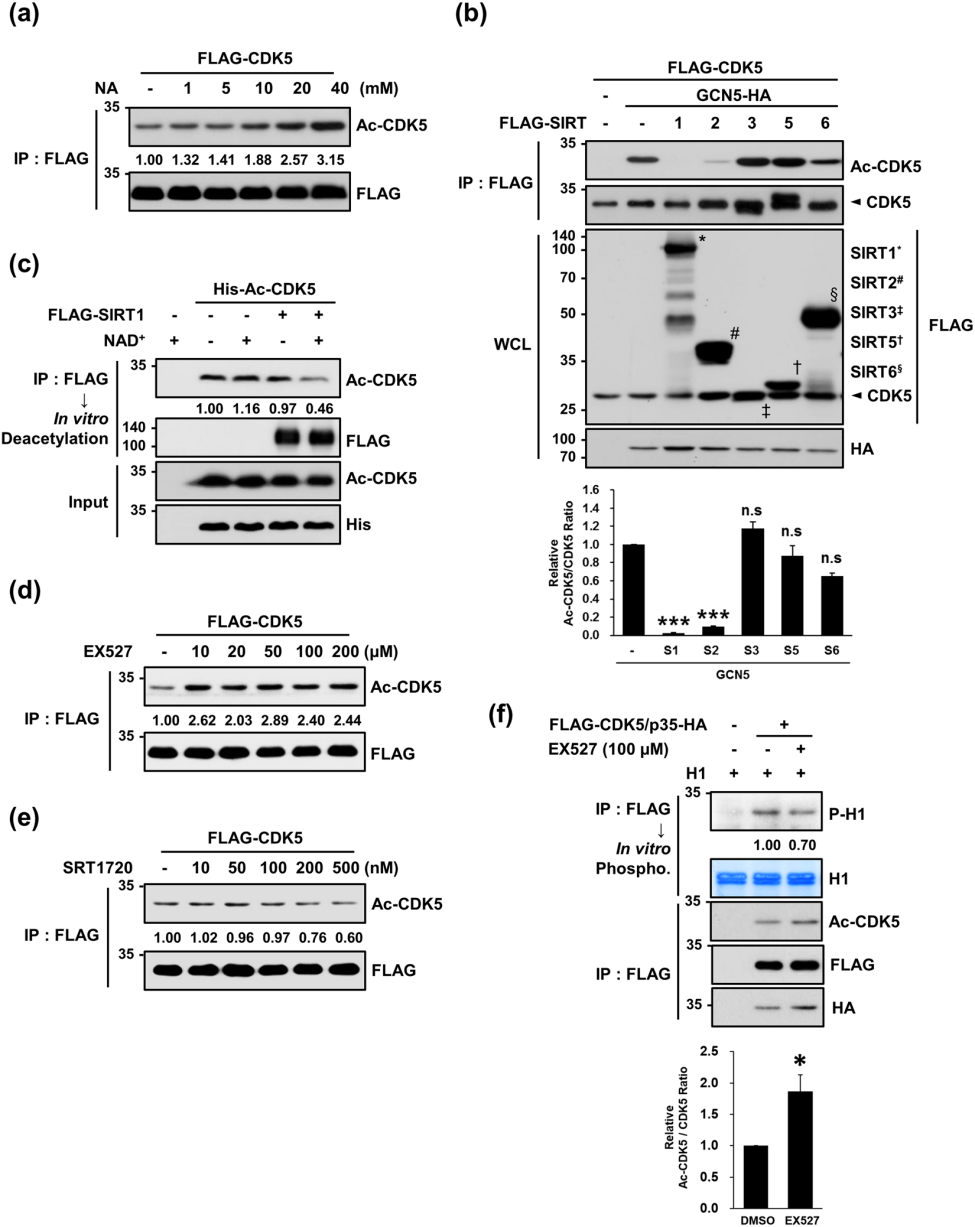
SIRT1 is responsible for the deacetylation of Ac-CDK5. (**a**) HEK293 cells transiently expressing FLAG-CDK5 were treated for 24 hrs with increasing doses of nicotinamide (NA, a pan-SIRT inhibitor). Lysates were subjected to IP with an anti-FLAG antibody and probed with the indicated antibodies. After normalization to FLAG-CDK5, the fold intensity of Ac-CDK5 versus control (value = 1) was determined. (**b**) HEK293 cells were transfected with one of the FLAG-tagged SIRTs plus FLAG-CDK5 and GCN5-HA. Lysates were subjected to IP with an anti-FLAG antibody and IB with an anti-Ac-CDK5 antibody. WCLs were subjected to IB with the indicated antibodies. Each band of SIRTs is marked by the indicated letters. After normalization to FLAG-CDK5, the fold intensity of Ac-CDK5 versus the control (value = 1) was indicated. The bar represents the mean ± S.D from three independent experiments. ^***^p < 0.001; n.s, not significant. (**c**) FLAG-SIRT1 was expressed in HEK293 cells and purified by IP with FLAG beads. SIRT1-bound beads were incubated with recombinant His-Ac-CDK5 supplemented with β-nicotinamide adenine dinucleotide (NAD^+^) to activate SIRT1. Reaction mixtures were subjected to IB with an anti-Ac-CDK5 antibody. The fold intensity of Ac-CDK5 versus the control (value = 1) was determined after normalization to the His-Ac-CDK5 inputs. The inputs were probed with the indicated antibodies. (**d**,**e**) HEK293 cells transfected with FLAG-CDK5 were treated with increasing doses of (**d**) EX527 (a selective SIRT1 inhibitor) or (**e**) SRT1720 (a selective SIRT1 activator) for 24 hrs. Lysates were subjected to IP with an anti-FLAG antibody followed by IB with an anti-Ac-CDK5 antibody. After normalization to FLAG-CDK5, the fold change over the control (value = 1) was determined. (**f**) Lysates were prepared from HEK293 cells transfected with FLAG-CDK5 and p35-HA and exposed to 100 μM EX527 for 24 hrs. Immunoprecipitates purified with an anti-FLAG antibody were subjected either to IB with the indicated antibodies or an *in vitro* phosphorylation assay in the presence of H1 and cold ATP. Phospho-H1 signals were visualized with an anti-phospho-H1 antibody. After normalization to FLAG-CDK5, the fold intensity of Ac-CDK5 versus the control (value = 1) was indicated. The bar represents the mean ± S.D from three independent experiments. ^*^p < 0.05.

Acetylation is regulated by a subset of deacetylases (HDACs) and is a critical modification step for numerous histone and non-histone proteins^30,31^. Thus, we experimentally searched for HDACs responsible for Ac-CDK5 deacetylation. First, CDK5-transfected HEK293 cells were treated with inhibitors targeting two distinct classes of HDAC: nicotinamide (NA, a nicotinamide adenine dinucleotide (NAD^+^)-dependent sirtuin family inhibitor) or trichostatin A (TSA, a broad-spectrum histone deacetylase class I and II inhibitor). Nicotinamide blocked a deacetylation step performed by sirtuins and consequently increased Ac-CDK5 levels in a dose-dependent manner (Fig. 3a). To identify the sirtuins responsible for the deacetylation of Ac-CDK5, HEK293 cells co-transfected with GCN5 and CDK5 plus one of the sirtuins were subjected to immunoblot analysis. Both SIRT1 and SIRT2 effectively deacetylated Ac-CDK5 (Fig. 3b). In contrast, the GCN5-mediated formation of Ac-CDK5 was not reversed and instead increased upon treatment with any of the HDACs tested (Supplementary Fig. 5b), although treatment with TSA over 500 nM increased CDK5 acetylation (Supplementary Fig. 5a). We then examined whether CDK5 interacts with SIRT1 and SIRT2. In reciprocal immunoprecipitation and immunoblot analyses with HEK293 cells, CDK5 interacted with SIRT1 and SIRT2 (Supplementary Fig. 6a,b). In *in vitro* deacetylation assays, Ac-CDK5 levels were markedly decreased by incubation with immunoprecipitated SIRT1 in the presence of NAD^+^ (Fig. 3c). Treatment of HEK293 cells with EX527 (a selective inhibitor of SIRT1) led to an increase in Ac-CDK5 levels, whereas SRT1720 (a selective activator of SIRT1) potentiated the deacetylation of Ac-CDK5 (Fig. 3d,e). Concomitantly increased Ac-CDK5 and decreased CDK5 kinase activities were detected in HEK293 cells upon exposure to EX527 (Fig. 3f). Similarly, an *in vitro* acetylation assay indicated that Ac-CDK5 levels decreased by SIRT2 expression in the presence of NAD^+^ (Supplementary Fig. 7).

### Temporal expression patterns of Ac-CDK5 in the developing rat cerebral cortices

**Figure 4:**
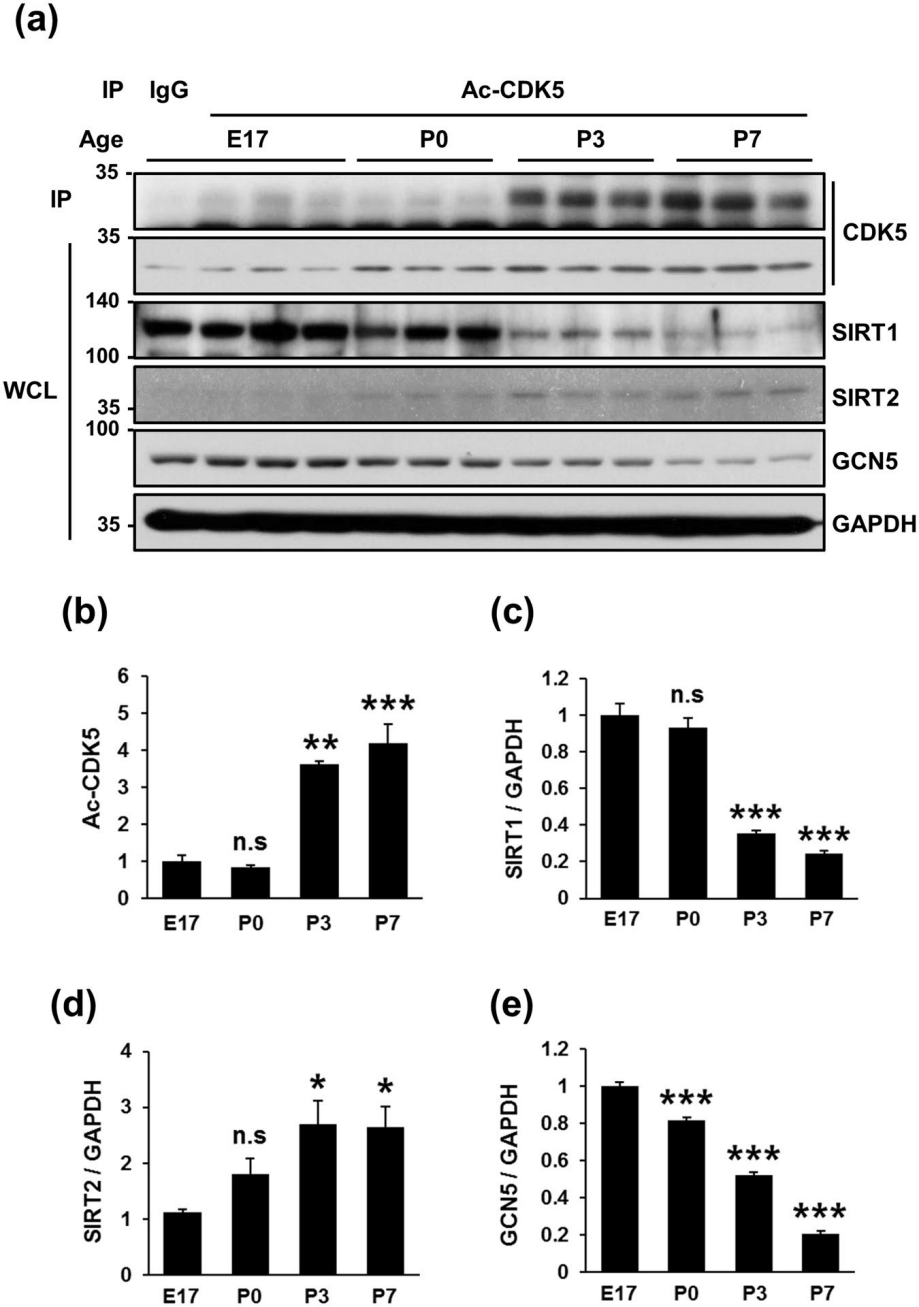
Temporal expression levels of Ac-CDK5 and SIRT1 are inversely correlated in the rat cortex. (**a**) Three tissue extracts obtained from rat cerebral cortices on embryonic day 17 (E17) and postnatal days 0 (P0), P3, and P7 were subjected to IP with an anti-Ac-CDK5 followed by IB with an anti-CDK5 antibody. CDK5, SIRT1, SIRT2 and GCN5 levels in WCLs were detected by IB with the corresponding antibodies. Blots represent one of three independent experiments. After normalization to GAPDH, relative levels were expressed as the fold intensity over E17 levels (value = 1). The bars represent the mean ± S.D of (**b**) Ac-CDK5, (**c**) SIRT1, (**d**) SIRT2 and (**e**) GCN5. ^***^p < 0.001; ^**^p < 0.01; ^*^p < 0.05; n.s, not significant.

To evaluate whether Ac-CDK5 plays a certain role during development, the temporal expression patterns of Ac-CDK5 in rat cerebral cortices were measured in relation to those of GCN5 and SIRTs. Very low levels of Ac-CDK5 were detected at embryonic day 17 (E17) and persisted until postnatal day 0 (P0) (Fig. 4a,b). Intriguingly, an abrupt increase in Ac-CDK5 expression was detected at P3 and maintained at P7. Higher levels of Ac-CDK5 were detected up to 8 weeks after birth (data not shown). The expression of CDK5 itself slightly increased over subsequent postnatal days (Fig. 4a). Conversely, SIRT1 expression levels were highest at E17 and maintained at P0 (Fig. 4a,c), followed by a dramatic decrease thereafter, demonstrating an inverse correlation with Ac-CDK5 levels. SIRT2 expression levels were lowest at E17 and increased thereafter as previously reported (Fig. 4a,d)^32^. GCN5 expression gradually decreased with age (Fig. 4a,e). Although we demonstrated above that both SIRT1 and SIRT2 deacetylate Ac-CDK5 in HEK293 cells, based on temporal expression patterns of these proteins in rat cerebral cortices, SIRT1 may act as a primary deacetylase to regulate Ac-CDK5 levels during early neural development.

### SIRT1-modulating drugs affect CDK5 kinase activity in primary cultures of embryonic hippocampal neurons

**Figure 5:**
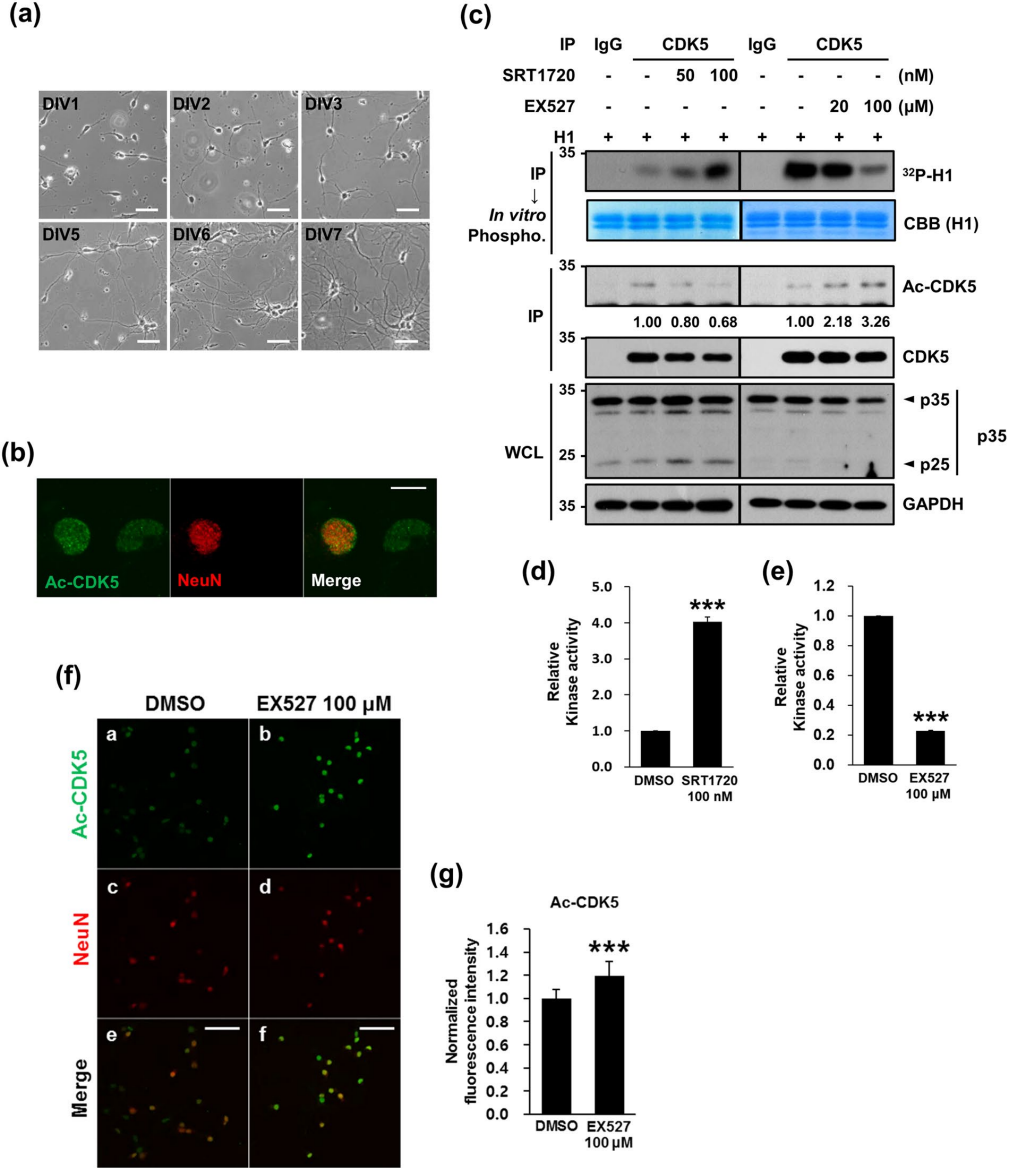
Pharmacological modulation of SIRT1 affects Ac-CDK5 levels and kinase activity in hippocampal neurons. (**a**) Primary cultures of hippocampal neurons were prepared from rat hippocampi isolated in gestational day 18. At the indicated days *in vitro* (DIV), photomicrographs were captured with an Axiovert 100. The scale bar represents 50 μm. (**b**) Cultured hippocampal neurons at DIV5 were fixed and immunostained with an anti-Ac-CDK5 antibody and an anti-NeuN antibody (a neuronal nuclear marker) followed by incubation with appropriate fluorescence-tagged secondary antibodies. Fluorescent images were obtained with an LSM700 confocal microscope. The scale bar represents 10 μm. (**c**) Hippocampal neurons at DIV3 were treated with SRT1720 or EX527 at the indicated doses for 48 hrs. Immunoprecipitates of cellular lysates purified with an anti-CDK5 antibody or IgG were subjected to IB with an anti-CDK5 antibody or an *in vitro* phosphorylation assay in the presence of H1 and [γ-^32^P]ATP. Phosphorylated H1 signals were visualized by autoradiography. After normalization to H1 or CDK5, the relative intensities of phospho-H1 and Ac-CDK5 were calculated over the controls (value = 1) and are indicated at the bottom of the blot. WCLs were subjected to IB with the indicated antibodies. (**d**,**e**) The relative kinase activity of CDK5 was expressed as the fold change over the control (value = 1) in the presence of (**d**) SRT1720 or (**e**) EX527. The bar represents the mean ± S.D from 4 independent experiments. ^***^p < 0.001. (**f**) Hippocampal neurons at DIV3 were treated with EX527 at 100 μM for 48 hrs, fixed, and then processed for immunofluorescent staining as described in (**b**). The scale bar represents 50 μm. (**g**) The fluorescence intensity of Ac-CDK5 in the nuclei of NeuN-positive neurons was measured using Image-J software. The relative fluorescence intensity was expressed as the fold change over the control (value = 1). The bars represent the mean ± S.D of 35 neurons from at least 5 randomly selected areas. ^***^p < 0.001.

CDK5 plays a pivotal role in neurite outgrowth, neuronal migration, and the regulation of synaptic development and function^2,10,12,16,17^. In addition, SIRT1 is involved in axon growth and dendritic arborization via the deacetylation of its substrates^33,34^. Given the overlapping effects of SIRT1 and CDK5 on neurite outgrowth, we examined whether the pharmacological regulation of SIRT1 activity affects the extent of neurite outgrowth by regulating the kinase activity of CDK5. We chose primary cultures of rat hippocampal neurons established from E18 embryos in which several hallmarks of neurite extension are easily monitored and reproducible^35^. In our cultures, the extent of neurite outgrowth was augmented over 7 days *in vitro* (DIV) (Fig. 5a). Immunocytochemistry revealed that nuclear puncta staining patterns of Ac-CDK5 were detected in both NeuN-positive and NeuN-negative cells, whereas staining of Ac-CDK5 in the cytosol was very faint (Fig. 5b). Using this model, we first determined whether EX527 (20–100 μM) and SRT1720 (50 and 100 nM) correspondingly altered Ac-CDK5 levels and CDK5 kinase activity. At the indicated concentrations, neither drugs caused any cytotoxicity as determined by counting cell numbers, soma size and signs of neurite fragmentation (data not shown). According to immunoprecipitation and immunoblot analyses, Ac-CDK5 levels decreased in a dose manner, whereas CDK5 kinase activity conversely increased in response to increasing doses of SRT1720 (Fig. 5c,d). In contrast, EX527 treatment led to increased levels of Ac-CDK5 and a concomitant decrease in kinase activity (Fig. 5c,e). Cleavage of p35 into p25 was slightly altered following drug treatment, but no significant correlation with CDK5 kinase activity was detected. Immunofluorescent localization revealed that the intensity of Ac-CDK5 nuclear staining was increased in both NeuN-positive and NeuN-negative cells after exposure to 100 μM of EX527 (Fig. 5f,g).

### CDK5 acetylation negatively regulates neurite outgrowth

**Figure 6:**
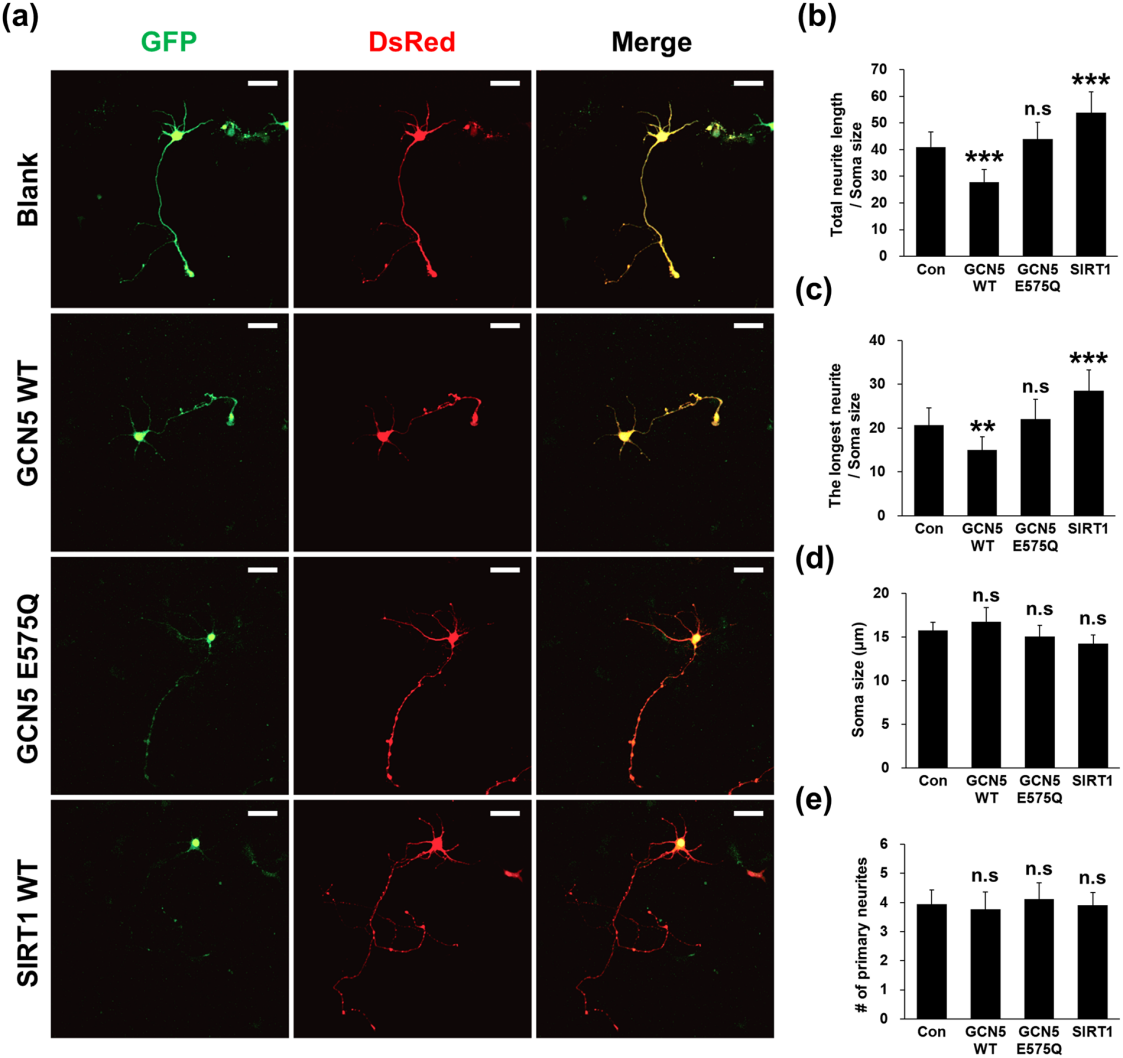
Overexpression of SIRT1 facilitates neurite outgrowth whereas overexpression of GCN5 retards neurite outgrowth in cultured hippocampal neurons. (**a**) Hippocampal neurons at DIV3 were transfected with either GFP-tagged GCN5 WT, GCN5 E575Q or SIRT1 WT along with DsRed vector. Fluorescent images were obtained with an LSM700 confocal microscope. The scale bar represents 50 μm. Confocal fluorescent images of (**b**) total neurite length, (**c**) the longest neurite length, (**d**) soma size and (**e**) the number of primary neurites were analyzed using Image-J software. The bars represent the mean ± S.D. of 35–40 neurons from at least 5 randomly selected areas. ^***^p < 0.001; ^**^p < 0.01; ns, not significant.

**Figure 7:**
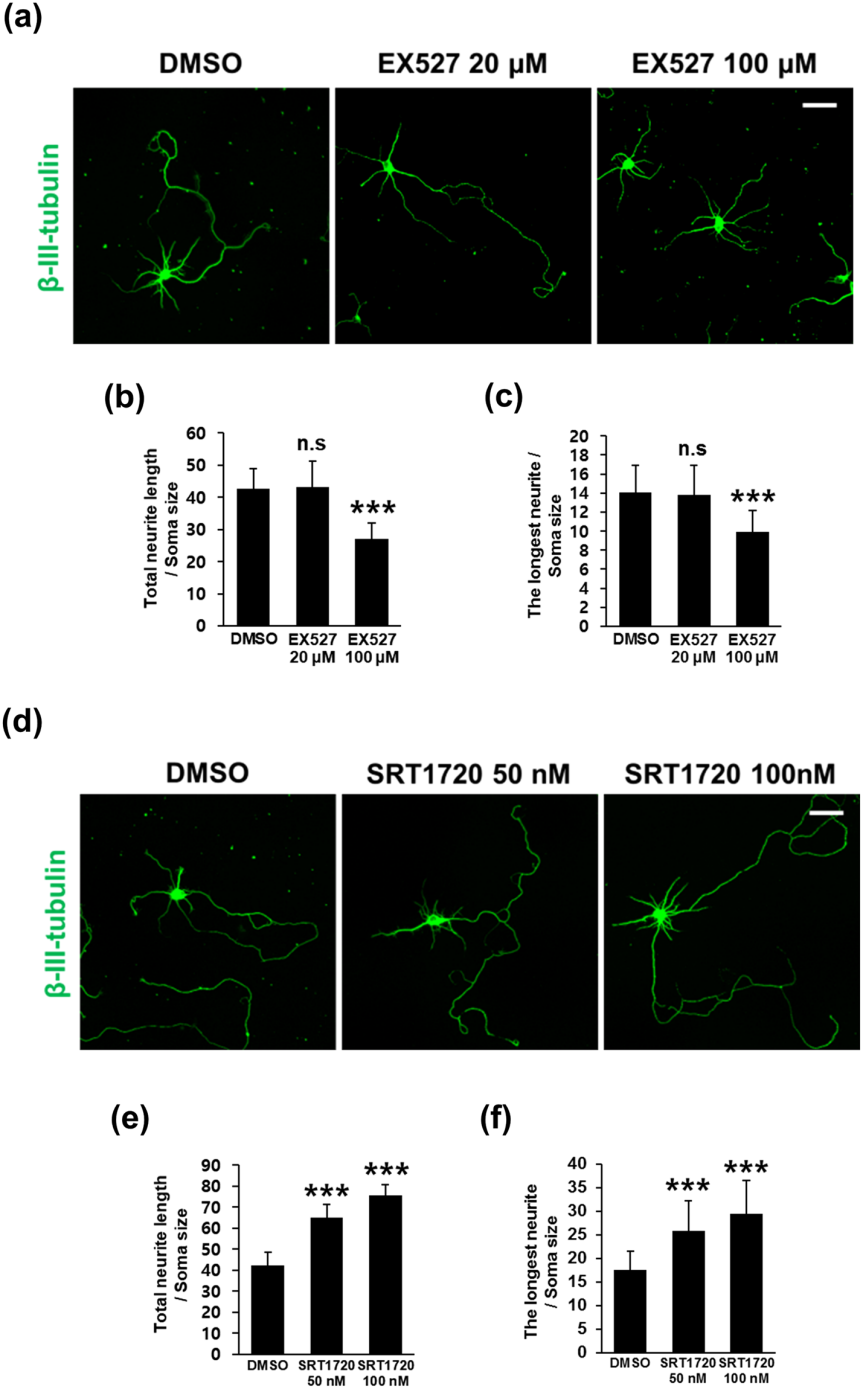
Pharmacological modulation of SIRT1 activity is linked to neurite outgrowth in cultured hippocampal neurons. Hippocampal neurons at DIV3 were treated with vehicle (DMSO) or (**a**–**c**) EX527 and (**d**–**f**) SRT1720 at the indicated doses for 48 hrs and subjected to immunofluorescent staining for neuron-specific β-III-tubulin. To measure the total neurite length and the length of the longest neurite, fluorescent images obtained with an LSM700 confocal microscope were analyzed using Image-J software. The scale bar represents 50 μm. Values are expressed as (**b**,**e**) the total neurite length and (**c**,**f**) the length of the longest neurite over the soma size. The bars represent the mean ± S.D. of 35–40 neurons from at least 5 randomly selected areas. ^***^p < 0.001; ns, not significant.

**Figure 8:**
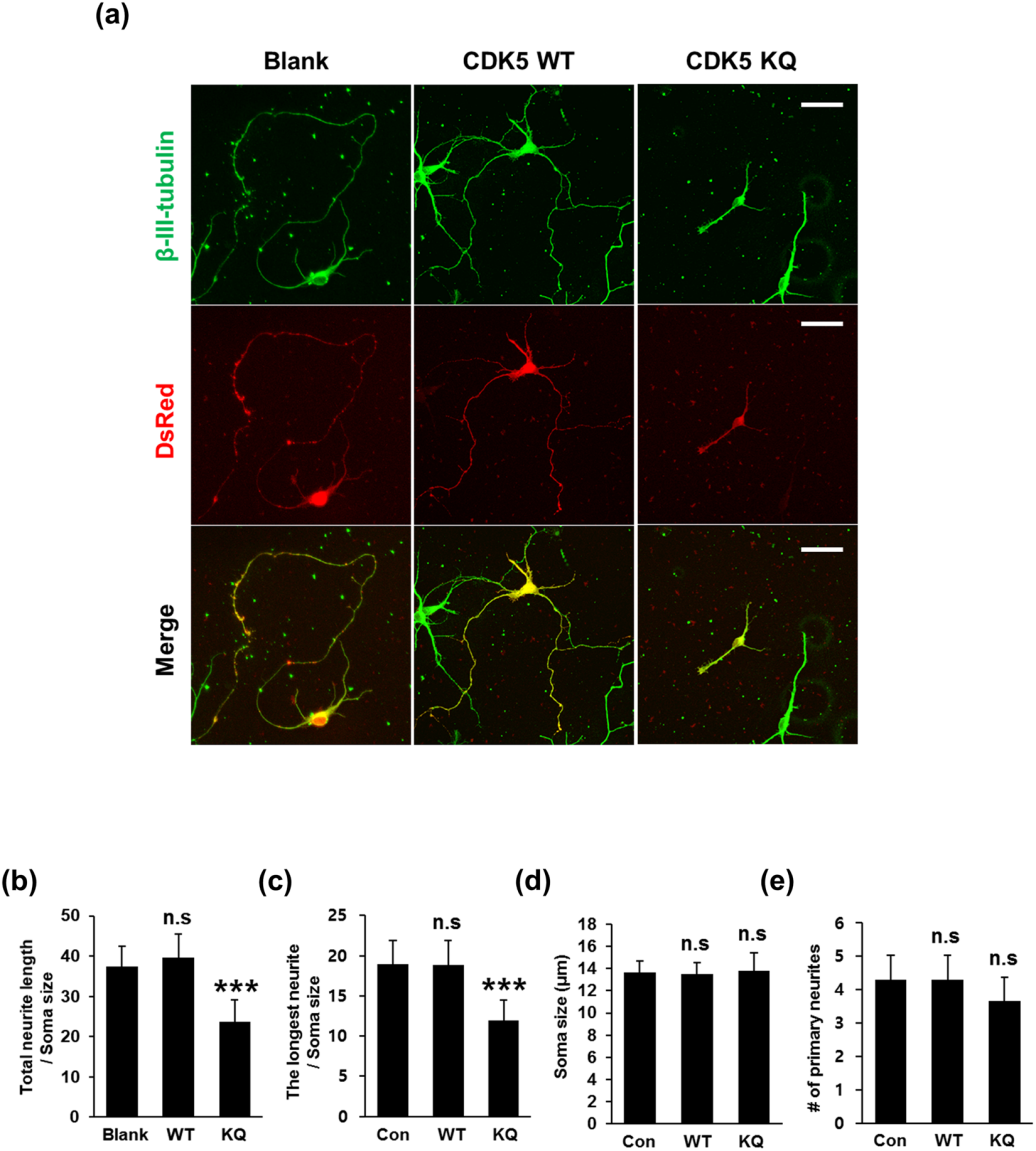
CDK5 acetylation hinders neurite outgrowth in hippocampal neurons. (**a**) Hippocampal neurons at DIV3 were transfected with a DsRed-expressing vector containing CDK5 WT or its K33Q mutant (CDK5 KQ) for 48 hrs and subjected to immunofluorescent staining for β-III-tubulin. Fluorescent images were obtained with an LSM700 confocal microscope. The scale bar represents 50 μm. Confocal fluorescent images of (**b**) total neurite length, (**c**) the longest neurite length, (**d**) soma size and (**e**) the number of primary neurites were analyzed using Image-J software. The bars represent the mean ± S.D. of 35–40 neurons from at least 5 randomly selected areas. ^***^p < 0.001; ns, not significant.

In accordance with our efforts seeking the relationship between CDK5 acetylation and neuron differentiation, we specifically attempted to reveal whether Ac-CDK5 levels affect the extent of neurite outgrowth in hippocampal neurons by independently running genetic and pharmacological analyses. First, ectopic expression of GCN5 WT in neurons revealed the retarded total neurite length and the longest neurite (Fig. 6a–c). This was in contrast to those found in GCN5 E575Q expressing neurons. Conversely, expression of SIRT1 accelerated total neurite length and the longest neurite. Both soma size and the number of primary neurites emanating from cell bodies were not affected in any conditions (Fig. 6d,e). We then tested the effect of SIRT1 activity-modulating drugs on neurite outgrowth. Treatment with 100 μM EX527 significantly impeded total neurite length and the length of the longest neurite (Fig. 7a–c). Conversely, SRT1720 led to enhancements in these two parameters (Fig. 7d–f). Neither EX527 nor SRT1720 affected primary neurite numbers or soma size (Supplementary Fig. 8a–d). To determine whether the SRT1720-mediated neurite elongation occurs in the CDK5-dependent manner, we measured changes of neurite length in neurons expressing shRNA for CDK5 in the presence or the absence of SRT1720 (Supplementary Fig. 9a,b). Interestingly, 20–30% knock down of CDK5 in protein levels did not affect the total neurite length as compared to that in neurons expressing control shRNA. However, SRT1720-mediated neurite elongation was not detected in two independent CDK5 knockdown neurons (Supplementary Fig. 9c,d), suggesting that CDK5 level itself is critically involved in determining extent of SRT1720-mediated neurite extension. Retinoic acid (RA) is a defined signaling molecule involved in early developmental processes, including neuronal patterning and neural differentiation^36^. In the SH-SY5Y neuroblastoma cell line, RA induces cell cycle arrest and neural differentiation, and therefore it has been adopted as a simple experimental system to study molecular signaling pathways associated with neuronal differentiation^37^. Treatment with 10 μM RA caused dramatic neurite extension (Supplementary Fig. 10a). Based on a protocol previously described (Supplementary Fig. 10b)^38^, the total neurite lengths of SH-SY5Y cells following treatment with RA plus SRT1720 or EX527 were compared. In support of SIRT1-induced changes in neurite length in hippocampal neurons, GFP fluorescence analysis revealed that SRT1720 further enhanced total neurite length evoked by RA treatment, whereas EX527-treated cells demonstrated the opposite outcome in SH-SY5Y cells (Supplementary Fig. 10c,d). Next, to directly ask the relationship between CDK5 acetylation and neurite outgrowth, the neurite outgrowth of hippocampal neurons transfected with the acetyl mimetic CDK5 K33Q mutant was measured. Overexpression of CDK5 WT itself did not cause any detectable alterations in morphology compared with mock-transfected hippocampal neurons. However, hippocampal neurons overexpressing CDK5 K33Q exhibited total neurite lengths and longest neurite lengths that were shortened (Fig. 8a–c), indicating that Ac-CDK5 levels negatively determine neurite length. No significant alterations were detected in soma size or the number of primary neurites in all groups (Fig. 8d,e). In a separate study, we evaluated whether pharmacological regulation of SIRT2 also affects neurite extension. Treatment with empirically determined doses of AGK2 (1 μM or 2.5 μM, SIRT2 inhibitor) resulted in a slight increase or decrease in Ac-CDK5 levels, respectively (Supplementary Fig. 11a,b). However, neither dose affected CDK5 kinase activity (Supplementary Fig. 11c). Under these conditions, AGK2 significantly increased total neurite length and the length of the longest neurite in hippocampal neurons (Supplementary Fig. 11d–h). Based on these data, SIRT2-mediated regulation of neurite length is independent of Ac-CDK5 levels and CDK5 kinase activity.

### Nuclear Ac-CDK5 determines neurite length

**Figure 9:**
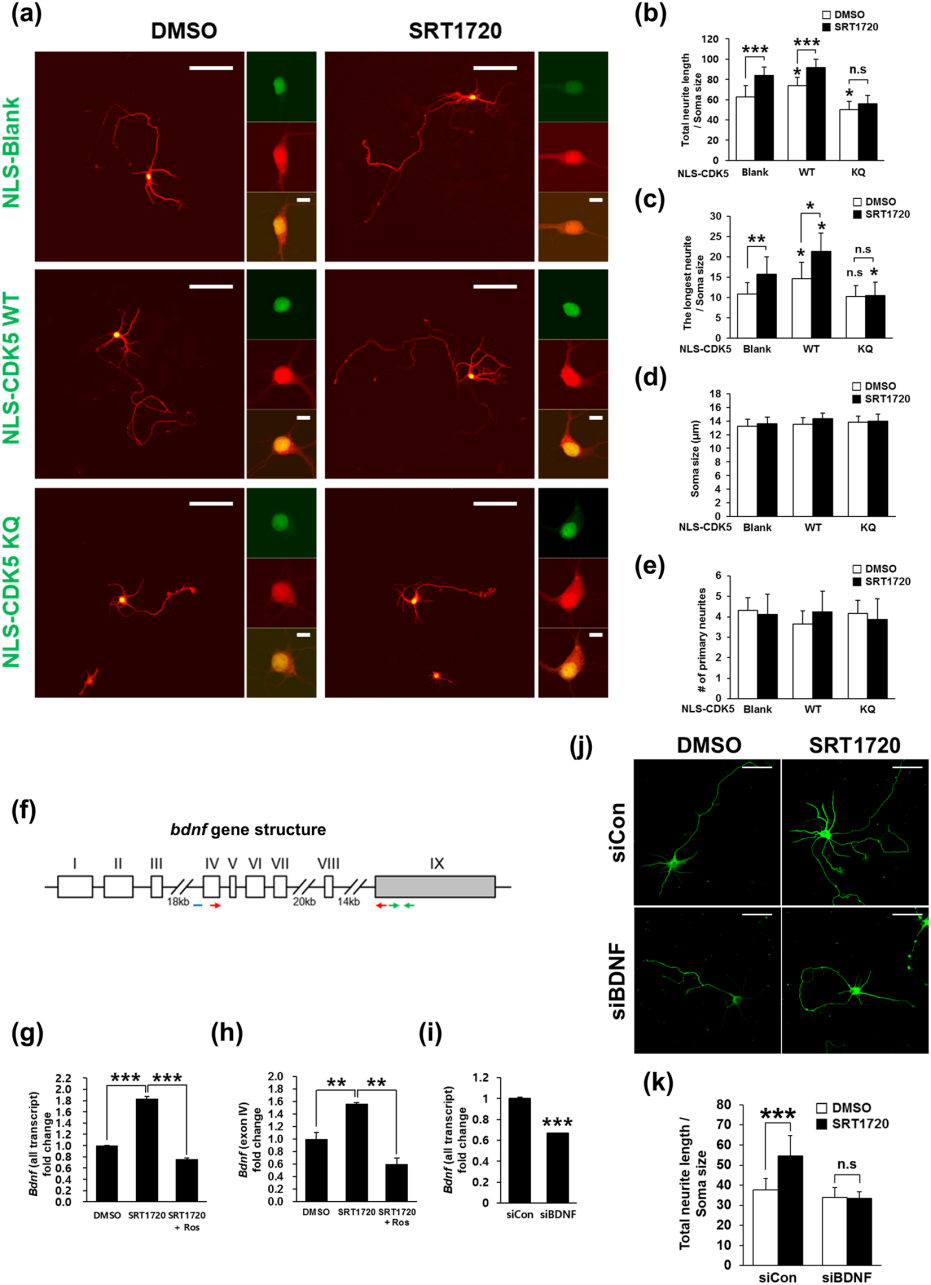
SIRT1-mediated deacetylation of nuclear CDK5 stimulates neurite outgrowth via upregulation of *BDNF* transcription. (**a**) Hippocampal neurons at DIV3 were transfected with nuclear localization signal (NLS)-GFP-CDK5 WT or NLS-GFP-K33Q and the DsRed vector. The cultures were then treated with vehicle or 100 nM SRT1720 for 48 hrs, fixed and imaged with a confocal microscope. Other photomicrographs show enlarged views with scale bars of 10 μm. Confocal images of (**b**) total neurite length, (**c**) the length of the longest neurite, (**d**) soma size and (**e**) the number of primary neurites were analyzed using Image-J software. The bars represent the mean ± S.D. based on the measurement of 35–40 neurons from at least 5 randomly selected areas. ^***^p < 0.001; ^**^p < 0.01; ^*^p < 0.05; n.s, not significant. The scale bar represents 50 μm. (**f**) Schematic diagram of the rat *bdnf* gene structure. *Bdnf* transcription is initiated by multiple promoters located upstream of distinct 5′ exons (white boxes, exon I to VIII) to give rise to transcript variants linked to the 3′ exon (gray box, exon IX). The real-time PCR primers for all *bdnf* transcripts and transcript containing exon IV are indicated by red and green arrows repectively. The location of the ChIP probe is indicated by a blue bar. (**g**,**h**) Hippocampal neurons at DIV3 were treated with 100 nM SRT1720 for 24 hrs followed by the addition of 10 μM roscovitine (Ros) for an additional 24 hrs. Total RNA was extracted from the cultures, reverse transcribed and subjected to quantitative real-time PCR for (**g**) all *bdnf* transcripts and (**h**) transcripts containing exon IV. The values are expressed as the fold-change over the control (value = 1). The bars represent the mean ± S.D. of three independent experiments. ^**^p < 0.01. (**i**) Total RNA from hippocampal neurons transfected with control siRNA or siRNA against *bdnf* were analyzed for levels of bdnf transcript by using quantitative real-time PCR. (**j**) The *bdnf*-knockdowned neurons or control siRNA-transfected neurons were treated with or without 100 nM SRT1720 for 48 hrs. Immunostaining with β-III-tubulin was performed to visualize neurite extension. The scale bar represents 50 μm. (**k**) Confocal fluorescent images of total neurite length were analyzed using Image-J software. The bars represent the mean ± S.D. based on the measurement of 35–40 neurons from at least 5 randomly selected areas. ^***^p < 0.001; n.s, not significant.

Based on our findings that (i) the endogenous Ac-CDK5 is strictly accumulated in the nucleus (Fig. 5b) and (ii) subcellular localization of endogenous CDK5 was not affected by GCN5 overexpression or EX527 treatment in HEK293 cells and hippocampal neurons (data not shown), we specifically attempted to elucidate the role of nuclear CDK5 acetylation in SIRT1-mediated neurite elongation. Hippocampal neurons were transfected with CDK5 WT and CDK5 K33Q subcloned into a nuclear targeting vector harboring additional nuclear targeting signals (NLS). Increases in total neurite length and longest neurite length were detected in NLS-CDK5 WT-transfected neurons, whereas overexpression of NLS-CDK5 K33Q led to a shortened total neurite length compared with that observed for mock-transfected neurons (Fig. 9a–c). Although it was not reached to the significant difference, a slight decrease in longest neurite length was detected in NLS-CDK5 K33Q-transfected neurons (Fig. 9c). Treatment with SRT1720 induced a further increase in total neurite length and longest neurite length in mock- or NLS-CDK5 WT-transfected neurons, indicating that CDK5 deacetylation is required for SRT1720-mediated neurite outgrowth. In contrast, SRT1720 did not cause any significant changes in the neurite lengths of NLS-CDK5 K33Q-transfected neurons. No changes in soma size or in the number of primary neurites were detected in all groups regardless of SRT1720 treatment (Fig. 9d,e). Since ectopically expressed CDK5 K33Q was localized in the cytosol as well as in the nucleus (Fig. 8a), we examined whether the cytosolic Ac-CDK5 may be involved in regulating neurite elongation of hippocampal neurons. To do so, we first examined whether treatment of hippocampal neurons with SRT1720 or EX527 affects the phosphorylation status of the well-known cytosolic substrates for CDK5. No discernible alteration in level of Tau and CHIP was detected regardless of drug treatment (Supplementary Fig. 12). Secondly, hippocampal neurons were transfected with the vector containing CDK5 K33Q sequences tagged with a nuclear export signal (NES). Forceful localization of CDK5 K33Q into the cytosolic region failed to inhibit neurite outgrowth (Supplementary Figure 13). Unexpectedly, total neurite length was rather increased in NES-CDK5 K33Q-expressing neurons (Supplementary Fig. 13b). As expected, no difference was observed in soma size and number of primary neurites (Supplementary Fig. 13d,e). Taken together, nuclear CDK5 kinase activity is required to neurite extension, which can be modulated by SIRT1-mediated deacetylation of CDK5 at K33.

### SRT1720-mediated activation of CDK5 induces *bdnf* expression

Neurotrophins comprise a family of proteins that are essential for neuronal survival, differentiation and function^39,40^. Recent studies have demonstrated that CDK5 is involved in brain-derived neurotrophic factor (BDNF)-induced dendritic growth^12^ and accumulates in the nucleus upon KCl-induced depolarization of cultured neurons to increase phosphorylation of methyl-CpG-binding protein 2 (MeCP2) at S421, which is released from its repressive association with the *bdnf* promoter^14,41^. Thus, we tested whether *bdnf* expression patterns correlate with SRT1720-triggered increases in neurite length and CDK5 kinase activity. The expression of either all *bdnf* transcripts or *bdnf* mRNA containing exon IV (Fig. 9f), which is repressively regulated by MeCP2, was significantly increased in hippocampal neurons treated with SRT1720, and this effect was abolished by roscovitine, a potent and selective CDK5 inhibitor (Fig. 9g,h). To investigate whether increased *bdnf* expression contributes SRT1720-mediated enhancement of neurite elongation, we transfected hippocampal neurons with siRNA against *bdnf*. Significant knockdown of *bdnf* was achieved but any sign of cell death was observed (Fig. 9i,j). Under this condition, we found that SRT1720-mediated increase in total neurite length was not detected in hippocampal neurons transfected with siBDNF (Fig. 9j,k), indicating *bdnf* expression is a downstream of SIRT1-induced nuclear CDK5 activation to execute neurite outgrowth. Next, we examined whether MeCP2 phosphorylation is affected by SRT1720 treatment. S421 phosphorylation in hippocampal neurons is not affected by SRT1720 treatment regardless of co-treatment with roscovitine (Supplementary Fig. 14a). Similarly, in chromatin immunoprecipitation (ChIP) assays employing primary cultures of cortical neurons, the association of MeCP2 with the *bdnf* exon IV promoter was not altered in any group (Supplementary Fig. 14b). Furthermore, MeCP2 phosphorylation at S80, which is a proline-directed residue required for chromatin association^42^, and activating phosphorylation of cAMP response element (CRE) binding protein (CREB) at S133^43^, a transcriptional activator of *bdnf* were not affected (Supplementary Fig. 14a), indicating that the CDK5 activity-dependent increase of neurite length was mediated by enhanced transcriptional regulation of BDNF via unknown mechanism(s) independent of MeCP2 and CREB. In sum, our study suggests that (i) SIRT1 plays a crucial role in determining neurite extension by regulating deacetylation-dependent CDK5 activity in the nucleus, and (ii) neurite extension can be achieved by upregulation of *bdnf* expression via CDK5′s phosphorylating activity-dependent mechanism(s) in embryonic hippocampal neurons (Supplementary Fig. 15).

## Discussion

Initial neuronal development begins with neurite extension followed by axon specification, dendritic branching and synapse formation. Our present study unravels a biochemical mechanism to explain how SIRT1 determines neurite outgrowth by modulating CDK5 activity via deacetylation in differentiating neurons. The acetylation of CDK5 at K33, a residue comprising the ATP binding pocket, induces a defect in kinase activity attributable to impaired ATP binding and is reversed by the deacetylation activities of SIRT1 and SIRT2. The K33 residue of CDK5 is structurally conserved between the CDK family, the mitogen-activated protein kinase (MAPK) family, and the AMP-activated protein kinase (AMPK) family. Indeed, a subset of these kinases including CDK2, CDK9, p38 and salt-inducible kinase 2 (SIK2), undergoes acetylation at positions equivalent to CDK5 K33^44–47^. Interestingly, p38 acetylation augments its kinase activity by increasing its ATP binding affinity^45^, unlike the SIK, CDK2, CDK9 and CDK5 kinases; thus, acetylation induces distinct effects, depending on the structural environment surrounding the catalytic ATP binding pocket. Together, these data strongly suggest that acetylation at the ATP binding site of CDK5-related kinases may be a crucial mechanism to direct overall kinase activity. Additionally, CDK5 activity under physiological conditions is negatively regulated by S-nitrosylation at C83, leading to inhibited dendritic growth among cultured hippocampal neurons^24^. Notably, C83 is a critical residue that comprises an ATP binding pocket similar to K33. Protein modifications that target the catalytic domain of CDK5 may represent an evolutionarily conserved mechanism to regulate kinase activity regardless of activator binding.

We consider SIRT1, but not SIRT2, an upstream deacetylase of CDK5 because SIRT1 expression levels are inversely correlated with CDK5 acetylation levels in developing brain, although SIRT2 also effectively deacetylates CDK5 in HEK293 cells. In contrast, our data and a previous study revealed that SIRT2 is maintained at lower levels during development and exponentially accumulates after the juvenile phase (3 weeks in rats, data not shown)^32^. Supporting this notion, SIRT1 is predominantly present in the nucleus in proximity to Ac-CDK5, whereas SIRT2 preferentially resides in the cytosol, although both can shuttle between the cytosol and the nucleus under different cellular circumstances, such as during differentiation, the cell cycle or apoptosis^48–50^. Furthermore, pharmacological inhibition of SIRT1 stimulates the accumulation of Ac-CDK5 in the nuclei of hippocampal neurons. Conversely, SIRT1 activation increases neurite length in association with increased CDK5 deacetylation. This effect is abolished by either CDK5 knockdown or the overexpression of nuclear-targeted acetyl-mimetic CDK5, indicating that SIRT1-mediated deacetylation of nuclear CDK5 at K33 is essential for neurite outgrowth in differentiating neurons. Therefore, our findings reveal a crucial role for nuclear CDK5, which is regulated by its (de)acetylation status via SIRT1 activity. Interestingly, SIRT1 itself promotes neuritogenesis and dendritic arborization in differentiating neurons. For example, SIRT1 overexpression further stimulates NGF-induced neurite outgrowth in PC12 cells^51^, and treatment with resveratrol, a natural SIRT1 activator, promotes neuritogenesis and neurite elongation in hippocampal neurons^34^. The mechanism underlying SIRT1-mediated neurogenesis involve protein kinase signaling pathways such as Akt, Rho-associated protein kinase (ROCK) and mammalian target of rapamycin (mTOR)^33,34,52^. Thus, SIRT1 contributes to normal neurogenesis by modulating differential phosphorylating activities of a subset of kinases, although further investigation is required to determine whether deacetylation of these kinases occurs directly via SIRT1.

How does SIRT1-activated nuclear CDK5 affect neurite outgrowth? SRT1720-mediated neurite outgrowth is accompanied by increased mRNA levels of *bdnf*, a neurotrophic factor that activates TrkB-PI3K/MAPK cascade in a cell-non-autonomous fashion^39^. The increased *bdnf* transcript levels are dramatically abolished by co-treatment with the CDK5 inhibitor roscovitine, suggesting that CDK5 functions downstream of SIRT1 to induce *bdnf* transcription. Although we could not detect a correlation between changes in phosphorylated MeCP2 or CREB levels and altered *bdnf* transcription, other cis-elements on the *bdnf* exon IV promoter are able to recognize transcription factors. These cis-elements include calcium responsive factor (CaRF), upstream stimulatory factor (USF) 1/2, nuclear factor κB (NF-κb) and NFATc4^41,53,54^. However, we do not exclude the possibility that *bdnf* mRNA may be regulated in a transcription independent manner including an alteration of its stability or editing^55^. Therefore, it would be of great interest to identify transcriptional regulators responsive to CDK5 activation and its associated pathways and determine whether SIRT1-mediated nuclear CDK5 deacetylation and the subsequent phosphorylation of one or more unidentified factor participate in neurite outgrowth via autocrine or paracrine actions of *bdnf*.

Because acetylation directly targets the critical residue for ATP binding to CDK5, modified CDK5 is fully “locked” to prevent activation, even after p35 is cleaved into the potent activator p25 when neurons are exposed to pathological conditions. Given its temporal expression pattern, SIRT2 may be the major deacetylating enzyme for CDK5 in aged brains. Furthermore, Ac-CDK5 in aged brains is maintained at substantially higher levels than in embryonic brains, indicating that acetylation may be critical to regulate CDK5 and avoid excessive activation in the nucleus. Although it is unclear whether nuclear SIRT2 levels are sufficient to affect CDK5 acetylation status under these conditions, it would be intriguing to explore whether the aberrant activation of CDK5 in aging-related neuronal disorders is modulated by the inhibition of SIRT2 activity. In this context, SIRT2 inhibition exerts beneficial effects on neurodegeneration. For example, the SIRT2 inhibitor AGK2 effectively rescues neurons from α-synuclein-induced neurotoxicity in Parkinson’s disease models^56^. SIRT1 is recruited to DNA double strand break sites and is required to stabilize HDAC1 and activate ATM autophosphorylation at S1981 to ameliorate DNA damage in cultured neurons^57^. Intriguingly, CDK5 likely participates in this process because CDK5-mediated ATM phosphorylation at S794 precedes and is crucial for ATM autophosphorylation upon DNA damage^58^. It would be of great interest to investigate whether CDK5 (de)acetylation is involved in SIRT1/2-mediated signaling in various neurodegenerative diseases characterized by aberrant CDK5 activation^8^, and to determine whether the modulation of acetylated CDK5 levels is a viable therapeutic target.

In conclusion, our findings provide evidence supporting a novel regulatory mechanism for nuclear CDK5 activity via (de)acetylation and the emerging role of SIRT1 as a determinant in this process. Given the broad spectrum of CDK5 action in neurons under physiological and pathological conditions, our findings lay the foundation to further understand brain development and the etiology of neurological disorders.

## Methods

### Cell cultures, drug treatments and transfections

Human embryonic kidney 293 (HEK293) cells originally purchased from the ATCC were routinely tested for mycoplasma contamination and independently maintained in the laboratory. HEK293 cells were cultivated in DMEM (GenDEPOT, #CM001) supplemented with 10% heat-inactivated FBS (GenDEPOT, #F0600) at 37 °C in a 5% CO_2_ atmosphere. For transient transfections, predetermined amounts of a eukaryotic expression plasmid were used to transfect HEK293 cells in combination with polyethylenimine (Sigma, #408727) as previously described^25^. Twenty-four hours after transfection, cells were treated with a pharmacological reagent for an additional 24 hrs. These reagents included nicotinamide (NA; Sigma, #N0636), trichostatin A (TSA; Sigma, T8552), SRT1720 (Santa Cruz Biotechnology, #sc-364624), EX-527 (Sigma, #E7034) and AGK2 (Sigma, #A8231). Primary cultures of hippocampal or cortical neurons were prepared from hippocampi or cerebral cortices derived form E17-18 Sprague-Dawley (SD) rat embryos as previously described with certain modifications^59^. Briefly, dissociated neurons were plated at a density of 5 × 10^3^ cells/cm^2^ on 100 μg/ml poly-D-lysine (Sigma, #P0899) and 1 μg/ml laminin (Invitrogen, #23017015)-coated glass coverslips in MEM supplemented with 0.6% D-glucose (Gibco, #15023021), 1 mM sodium pyruvate (Sigma, #P5280), 2 mM L-glutamine (Sigma, #G8540), 100 U/ml penicillin-streptomycin (Invitrogen, #15140122) and 10% FBS (Gibco, #26140). Twenty-four hours after plating, the medium was changed to a neurobasal medium (Invitrogen, #21103049) supplemented with 2% B27 (Invitrogen, #175040) and 0.5 mM L-glutamine. Phase-contrast photomicrographs were captured with a Zeiss Axiovert 100 microscope (Carl Zeiss). Hippocampal neurons at DIV3 were treated with SRT1720 or Ex527 for 48 hrs. For transient expression studies, hippocampal neurons at DIV3 were transfected with the indicated plasmids using Lipofectamine 2000 (Invitrogen, #11668019) according to the manufacturer’s protocol. All experimental animal procedures were in accordance with the National Institutes of Health Guide for the Care and Use of Laboratory Animals and approved by the Institutional Animal Care and Use Committees of Yonsei University.

### Plasmid construction, mutagenesis and PCR

To carry out the epitope-tagging of desired proteins, FLAG, HA and Myc sequences were inserted into the Nhe1 site of a pCI-neo mammalian expression vector (Promega, #E1841). Mouse CDK5, p25 and p35 sequences were generated by PCR. All constructs harboring CDK5, p35 or p25 vectors were generated via subcloning into one of these modified vectors. The K33R and K33Q mutants of mouse CDK5 were generated using a site-directed mutagenesis kit (Agilent Technologies, #200518). DsRed-tagged mouse CDK5 WT and its mutants were constructed by inserting PCR products into the pDsRed2-N1 vector (Clontech, #632406). FLAG-tagged human PCAF and HA-tagged human HBO1 were provided by Dr. Hyunsook Lee of Seoul National University and by Dr. Jacques Côte of Laval University, respectively. Protein S-FLAG-streptavidin binding protein (SFP)-tagged human SIRT1 and SIRT6 were kindly provided by Dr. Ja-Eun Kim of Kyunghee University. All other plasmids containing KATs and SIRTs were purchased from Addgene (Cambridge). GFP-tagged human GCN5 was constructed by introducing the appropriate PCR product into the pAcGFP-C1 vector (Clontech, #632470). Nuclear localization signal (NLS) -tagged CDK5 WT and K33Q, and nuclear export signal (NES)-tagged CDK5 K33Q were generated by subcloning into the pEF/myc/nuc/GFP vectors (Invitrogen, #V89120). For knockdown experiments, shRNAs against rat CDK5 were cloned into pRNAT-U6.1/Neo (Genscript, #SD1211). The target sequences are: shCDK5#1, 5′-gaagctcacattggtgtttga-3′; shCDK5#2, 5′-gaatctgctcattaacaggaa-3′. siRNA against rat *bdnf* was purchased from Origene (#SR510549) and transfected into neurons using RNAiMAX (ThermoFisher, #13778) as recommended by the manufacturer’s protocol.

### Site-directed acetylation and purification of recombinant proteins

To produce a His-tagged recombinant CDK5 protein acetylated at K33 (abbreviated as Ac-CDK5 below), a method to genetically apply site-specific acetylation on recombinant proteins was performed as previously described (Supplementary Fig. 1a)^26^. The pCDF-PylT and pBK-AcKRS-3 vectors were generously provided by Dr. Jason W. Chin of the University of Cambridge. Briefly, *E*. *coli* was transformed with the pBK-AcRS3 vector encoding acetyl-lysyl-tRNA synthetase, and the pCDF-PylT vector harboring His-tagged mouse CDK5 mutated into the amber codon (TAG) at the K33 site and tRNA_CUA_. The native stop codon (TAG) of CDK5 was also mutated into TAA to avoid undesired acetylation. After IPTG induction, acetyl-lysine was incorporated into the K33 site to replace native lysine via recognition of the mutated amber codon by tRNA_CUA_. Nicotinamide (NA) was added to the culture to inhibit the endogenous bacterial deacetylase CobB. Recombinant His-tagged Ac-CDK5 and -CDK5 were purified with Ni-NTA beads, and the eluted protein was concentrated in 50 mM Tris (pH 7.5), 150 mM NaCl, 5% glycerol, 20 mM NA, and a protease inhibitor cocktail (PIC; Roche, #11873580) using an Amicon^®^ Ultra-0.5 ml centrifugal filter (Millipore, #UFC501096). To purify the His-tagged p25 protein, mouse p25 was cloned into a pET-28a(+) vector (Millipore, #69864) and purified as described above. All proteins were stored at −80 °C until use.

### Preparation of anti-Ac-CDK5 antibody

To generate a rabbit polyclonal antibody against Ac-CDK5, we designed the peptide EIVAL(acK)RVRLD, which corresponds to sequences surrounding the K33 residue within human CDK5. The antibody production process was performed by Abmart (Shanghai, China). Antibody specificity was determined as described in Supplementary Fig. 1 and 2. Several dilution ranges were employed (1:1,000 for immunoblot analysis; 1:100 for immunoprecipitation and 1:200 for immunocytochemistry).

### Immunoblot analysis and immunoprecipitation

Cellular lysate preparation and immunoblot analysis were performed as described^25^. To prepare tissue lysates, male rats of different ages were obtained from Orient (Seongnam-si, Gyeonggi-do, Korea) and euthanized in a CO_2_ chamber. Rat cerebral cortices were rapidly isolated and homogenized via sonication in RIPA buffer containing PIC. For these experiments, rats were randomized and investigators were not blinded. The nuclear fraction was prepared according to a protocol provided by Abcam. The following commercially available antibodies were used for immunoblot analysis and immunoprecipitation: mouse monoclonal anti-FLAG-peroxidase (M2; 1:3,000, Sigma, #A8592); anti-hemagglutinin (HA)-peroxidase (3F10; 1:1,000; Roche, #12013819001); anti-V5-peroxidase (1:5,000; Invitrogen, #R961-25); anti-GAPDH antibody (6C5; 1:4,000; Millipore, #MAB374); anti-GCN5 (C26A10; 1:1,000; Cell Signaling Technology, #3305); and rabbit polyclonal anti-acetylated-lysine (1:1,000; Cell Signaling Technology, #9441). All other antibodies were purchased from Santa Cruz Biotechnology, including rabbit polyclonal anti-CDK5 (C8; 1:1,000; #sc-173), anti-His probe (H15; 1:1,000; #sc-803), anti-SIRT1 (H-300; 1:1,000; #sc-15404), anti-SIRT2 (H-95; 1:1,000; #sc-20966), anti-p35 (C19; 1:1,000; #sc-820), mouse monoclonal anti-CDK5 (J3; 1:1,000; #sc-6247), anti-CHIP (G2; 1:1,000), and goat polyclonal anti-SOD-1 (C17; 1:1,000; #sc-8637) and anti-Lamin A/C (N18; 1:1,000; #sc-6215). Rabbit polyclonal anti-MeCP2 (1:1,000, ECM Bioscience, #MP4591), anti-phospho-MeCP2 (S421) (1:1,000; ECM Bioscience, #MP4611), anti-phospho-MeCP2 (S80) (1:1,000; ECM Bioscience, #MP4601), rabbit monoclonal anti-CREB (48H2; 1:1,000; Cell Signaling Technology, #9197) and anti-phospho-CREB (S133) (87G3; 1:1,000; Cell Signaling Technology, #9198) were also used. Mouse monoclonal antibodies against for total tau (Tau-1; 1:5,000) and paired helical filaments (PHF; 1:5,000) were kindly provided by Dr. Virginia M. Lee at University of Pennsylvania. Rabbit polyclonal antibody for phospho-CHIP (Ser20) was produced by Abmart^60^. Membranes were incubated at RT for 1 hr with HRP-conjugated goat anti-rabbit (1:5,000; Santa Cruz, #sc-2004), goat anti-mouse (1:5,000; Santa Cruz, #sc-2005) or donkey anti-goat antibody IgG (1:10,000; Abcam, #ab6885). For immunoprecipitation, protein (1–2 mg) was pre-incubated with protein A agarose beads (Millipore, #16–125) for pre-clearance and then further incubated with 1–2 µg of corresponding antibodies or 10 µl of anti-Flag M2 affinity gel beads (Sigma, #A2220) in RIPA buffer at 4 °C overnight. Immunocomplexes were collected with protein A agarose beads followed by centrifugation at 800 × g at 4 °C for 2 min. Proteins were eluted from the beads with 1 × protein sample buffer, denatured by boiling, separated by 11.5% SDS-PAGE and subjected to immunoblot analysis employing corresponding antibodies. Specific bands were detected with enhanced chemiluminescence (ECL; Amersham Bioscience, #NEL105). Band intensity was quantified using Image-J software provided by the National Institutes of Health (NIH). Original images of the uncropped blots are shown in the Supplementary material.

### Immunofluorescence microscopy

Immunofluorescence microscopy was performed with rabbit polyclonal anti-Ac-CDK5 (1:200), anti-neuron specific beta-III-tubulin (1:200; Abcam, #ab18207), and mouse monoclonal anti-NeuN (A60; 1:200; Millipore) antibodies. The following secondary antibodies were used: Alexa-488-conjugated goat anti-rabbit IgG (ab150077; 1:200; Abcam) or anti-mouse IgG (ab150117; 1:200; Abcam) and Alexa-594-conjugated goat anti-rabbit IgG (1:200; Abcam, #ab150084) or anti-mouse IgG (1:200; Abcam, #ab150120). Nuclei were counterstained with 2 μg/ml Hoechst 33258 (Invitrogen, #H3569). Glass coverslips were mounted on methanol-soaked glass slides with Vectashield mounting medium (Vector Laboratories, #H1000). Fluorescence images were observed under an LSM 700 confocal microscope equipped with epifluorescence and LSM image browser (Carl Zeiss).

### Pull-down assay (Ni-NTA)

Purified His-CDK5 WT or His-Ac-CDK5 (10 μg) diluted in PBS containing PIC and 10 mM NA was incubated with 30 μl of Ni-NTA resin (Invitrogen, #R901) at 4 °C overnight. Cell lysates (2 mg) transfected with p35 or p25-FLAG were pre-incubated with 30 μl of Ni-NTA resin at 4 °C for 2 hrs to avoid non-specific protein binding. The pre-cleared cell lysates were then incubated with His-CDK5-bound Ni-NTA resin at 4 °C overnight under gentle rotation. CDK5/p35 or p25 complexes were washed with PBS and subjected to SDS-PAGE followed by immunoblotting with antibodies targeting FLAG, His and Ac-CDK5.

### *In vitro* phosphorylation and deacetylation assays

*In vitro* phosphorylation assays were performed as previously described^60^. Briefly, purified recombinant His-CDK5 WT or His-Ac-CDK5 (1 µg) was incubated at 30 °C for 30 min with 1 µg of histone H1 protein (Merck, #382150) and 1 µg of p25-His in 1 × kinase buffer (40 mM Tris-HCl pH 7.6, 2 mM DTT, 5 mM MgCl_2_, 1 mM NaF, 50 µM unlabeled ATP) in the presence of 5 µCi [γ-^32^P]ATP or cold ATP. Phospho-H1 signals were visualized by autoradiography or immunoblotting with a rabbit polyclonal anti-phospho-histone H1 antibody (1:1,000; Millipore, #06-597). Transiently transfected HEK293 cells and hippocampal neurons were lysed, immunoprecipitated with anti-CDK5 antibody and processed for *in vitro* phosphorylation assays. To assess ATP binding affinity, purified recombinant His-CDK5 WT and His-Ac-CDK5 (1 μg) were reacted for 2 hrs with 20 μl of ATP-conjugated agarose (Jena Bioscience, #AC-101) as previously described^61^. For the *in vitro* deacetylation assay, immunoprecipitated FLAG-tagged SIRT1 or SIRT2 was incubated with 0.5 µg of purified recombinant Ac-CDK5 supplemented with 5 mM β-nicotinamide adenine dinucleotide (NAD^+^, Sigma, #N7004) at 30 °C for 2 hrs as previously described^62^.

### ATP binding assay

For fluorescence measurements^63–65^, purified CDK5 proteins (500 ng) were incubated with varying concentration of mant-ATP (2′/3′-O-(N-methylanthraniloyl)-adenosine-5′-triphosphate, triethylammonium salt, Jena Bioscience, #NU-202) in a binding buffer (50 mM Tris-HCl [pH 7.4], 150 mM NaCl, 0.1% BSA) at 37 °C. Mant-ATP binding to either CDK5 WT or Ac-CDK5 was monitored by following fluorescence at λex 355/λem 460 nm (threshold λ455 nm) on a FlexStation 3 multi-mode microplate reader (Molecular Devices). Nonlinear regression was performed to obtain a best-fit curve for a specific binding [Y = Bmax*X/(Kd + X)] to the fluorescence raw data using GraphPad Prism 7 (X, the varying concentration of mant-ATP; Y, relative fluorescence intensity of specific binding; Bmax, maximum specific binding, Kd, equilibrium binding constant; Binding potential = Bmax/Kd). Representative data were presented as the mean ± SEM from at least three to four independent biological replicate experiments with triplicate measurements. For testing the differences in specific binding to mant-ATP between CDK5 WT and Ac-CDK5, Wilcoxon matched-pairs rank test was employed, unless otherwise noted. The effectiveness of pairing was also analyzed by calculating nonparametric Spearman correlation coefficient. Data from CDK5 binding experiments using mant-ADP as well as mant-ATP were evaluated by one-way analysis of variance (Friedman test) with Dunn’s *post-hoc* analysis for multiple comparisons. Statistical calculations were carried out using Prism 7 (GraphPad). *P* < 0.05 was considered statistically significant.

### Measurement of neurite outgrowth

To evaluate neurite outgrowth in hippocampal neurons, more than 35 neurons were randomly selected from a minimum of 3–5 glass coverslips per experiment and imaged by Z-stacking by an individual who was blind to the treatment conditions. After image processing using LSM image browser, fluorescent signals from beta-III-tubulin, GFP- or Ds-Red-containing vectors were measured to assess total neurite length, the length of the longest neurite, the number of primary neurites and soma size using Image-J software. Only processes longer than the soma size were considered neurites. The number of primary neurites was defined as the number of neurite segments originating directly from the soma. As previously described^66^, the ratio of total neurite length or the length of longest neurite to soma size was determined to minimize the effects of soma size on neurite length. The representative experimental data were provided from at least 2–3 independent experiments performed.

### Real-time quantitative PCR and chromatin immunoprecipitation (ChIP)

Real-time PCR was performed using a SensiFAST SYBR Hi-Rox kit (Bioline, #BIO-92005) according to the manufacturer’s protocol. The following oligonucleotide primers were used for real-time PCR: rat BDNF (all transcript) sense, 5′-TTGAGCACGTGATCGAAGAG-3′, and antisense, 5′-CCAGCAGAAAGAGCAGAGGA-3′; rat BDNF (exon IV) sense, 5′-CTGCCTTGATGTTTACTTTGACAAG-3′, and antisense, 5′-ACCATAGTAAGGAAAAGGATGGTCAT-3′; rat GAPDH sense, 5′-GACATGCCGCCTGGAGAAAC-3′, and antisense, 5′-AGCCCAGGATGCCCTTTAGT-3′. For the ChIP assay, rat primary cortical neurons plated at 2 × 10^7^ cells per P-150 dish were treated at DIV 3 with 100 nM SRT1720 for 24 hrs followed by 10 μM roscovitine for an additional 24 hrs. As directed by the protocol provided by Santa Cruz Biotechnology, DNA-protein complexes were crosslinked, quenched, lysed and then immunoprecipitated with 5 μl of anti-MeCP2 antibody or rabbit IgG at 4 °C overnight, followed by incubation with 20 μl of protein A agarose for an additional 2 hrs. MeCP2/DNA complexes were eluted in 300 μl of elution buffer (1% SDS, 100 mM NaHCO_3_) at 65 °C overnight, reverse cross-linked by adding 5 μl of RNase A (iNtRON Biotechnology, #27062) and incubated for 4 hrs. The following PCR primers were used for the ChIP assay: BDNF promoter (exon IV) sense 5′- GCGCGGAATTCTGATTCTGGTAAT-3′, and antisense 5′-GAGAGGGCTCCACGCTGCCTTGACG-3′.

### Statistics

All data are expressed as the means ± S.D. Significance between groups was determined by performing one-way ANOVA and Tukey’s post hoc test using GraphPad Prism 6 (GraphPad software). Two-tailed Student’s *t*-tests were also used when necessary (Figs 5e and 8). Values of ^***^p < 0.001, ^**^p < 0.01, and ^*^p < 0.05 were considered significant.

## Electronic supplementary material

Supplementary information
