# Supplementary material for: The acetylation of cyclin-dependent kinase 5 at lysine 33 regulates kinase activity and neurite length in hippocampal neurons
Source: Sci Rep. 2018 Sep 12;8:13676. doi: 10.1038/s41598-018-31785-9 (PMC6135752; doi:10.1038/s41598-018-31785-9)

The acetylation of cyclin-dependent kinase 5 at lysine 33 regulates kinase activity and neurite length in hippocampal neurons

Juhyung Lee[1,2], Yeon Uk Ko[1], Yuhyun Chung[1], Nuri Yun[1], Myungjin Kim[3], Kyungjin Kim[3,4,*] and Young J. Oh[1,*]

Supplementary Table 1. Summary of the best-fit values of binding parameters between CDK5 WT and Ac-CDK5. Bmax, Kd and binding potential were shown.

	Bmax	Kd (μM)	Binding Potential (Bmax/Kd)
**CDK5 WT**	2805.69	791.67	3.54
**Ac-CDK5**	2274.84	837.23	2.72

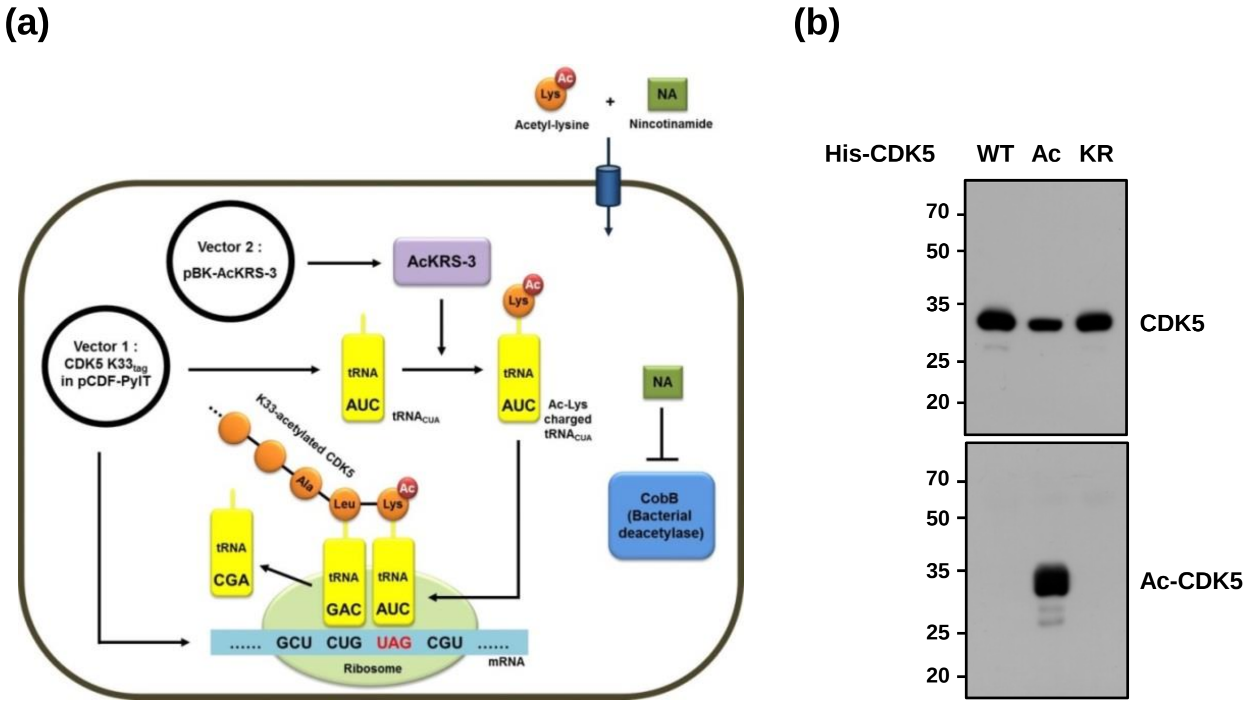

Supplementary Figure 1. Strategy for site-directed acetylation and purification of Ac- CDK5. (a) Schematic diagram depicting the genetically directed site-specific acetylation of recombinant proteins (see the Methods for more details). **(b)** To assess the purity, 100 ng of CDK5 WT, Ac-CDK5 (Ac) and CDK5 K33R (KR) were subjected to IB with an anti-CDK5 antibody (top blot) or anti-Ac-CDK5 antibody (bottom blot)

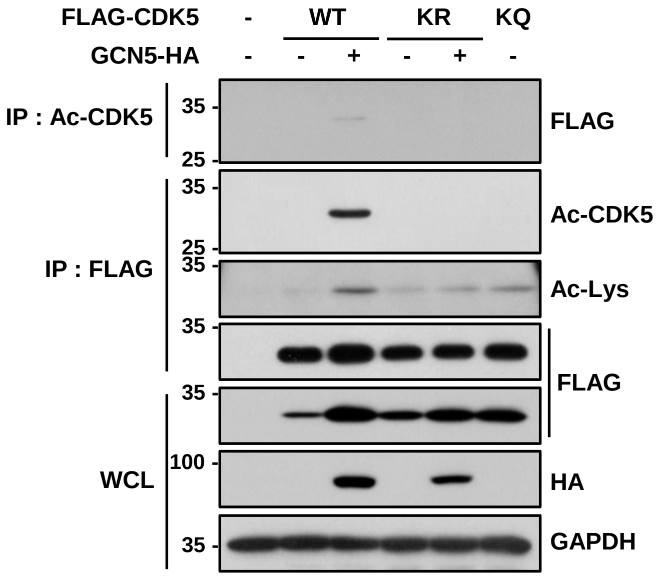

Supplementary Figure 2. Characterization of the rabbit polyclonal anti-Ac-CDK5 antibody. To raise an antibody against Ac-CDK5, rabbits were immunized with a designed peptide, EIVAL(acK)RVRLD, corresponding to the sequence surrounding the K33 residue within human CDK5. To characterize the generated rabbit polyclonal antibody, HEK293 cells were transfected with FLAG-CDK5 WT, -K33R (KR) or -K33Q (KQ) plus GCN5-HA. The lysates from each transfectant were subjected to IP with an anti-Ac-CDK5 antibody or anti-FLAG antibody followed by IB with the indicated antibody. Anti-acetylated-lysine antibody (Ac-Lys) was also employed for IB with FLAG-immunoprecipitates to measure total CDK5 acetylation levels. WCLs were subjected to IB with the indicated antibody to measure input levels.

(a)

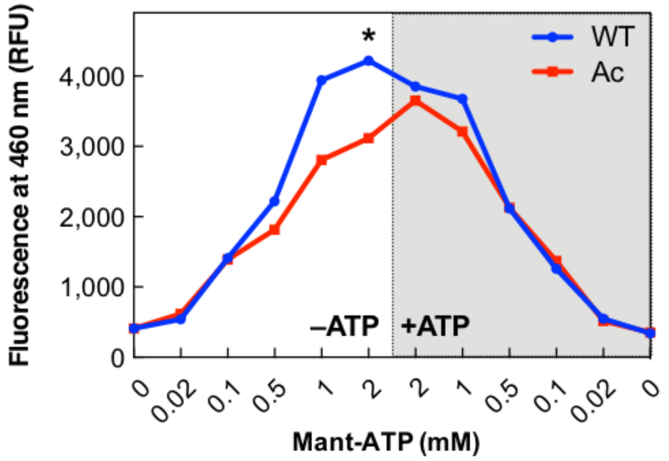

(b)

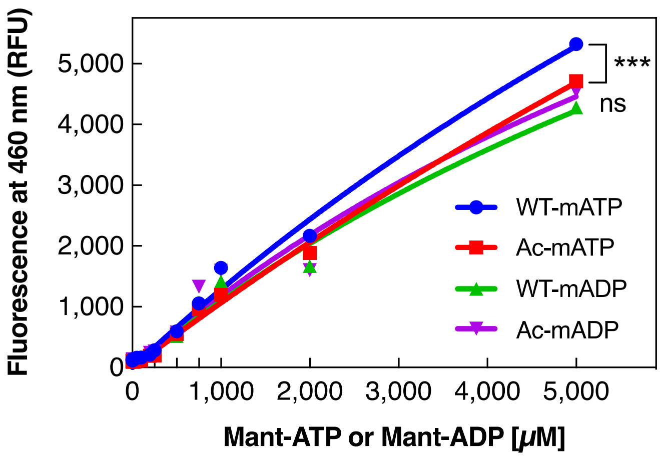

Supplementary Figure 3. Acetylation of CDK5 inhibits ATP binding. (a) Recombinant His-CDK5 WT (blue-filled circles) or His-Ac-CDK5 (red-filled rectangles) was incubated with increasing concentration of mant-ATP in the presence or absence of excess ATP (2 mM). Nonlinear regression was performed to obtain a best-fit curve for a specific binding. Paired *t*-test was employed to test the binding difference between CDK5 WT and Ac-CDK5 ($^{*}P$=0.03; correlation coefficient, r=0.976). **(b)** Recombinant His-CDK5 WT or His-Ac-CDK5 was incubated with increasing concentration of either mant-ATP or mant-ADP. Nonlinear regression was performed to obtain a best-fit curve for a specific binding. Friedman test was employed to evaluate the binding difference between CDK5 WT and Ac-CDK5 ($^{***}P$<0.001), followed by Dunn's *post hoc* analysis for multiple comparisons [$^{***}P$<0.001, WT-mATP (blue circle) vs. Ac-mATP (red square); ns (not significant), WT-mADP (green triangle) vs. Ac-mADP (purple inverted triangle)].

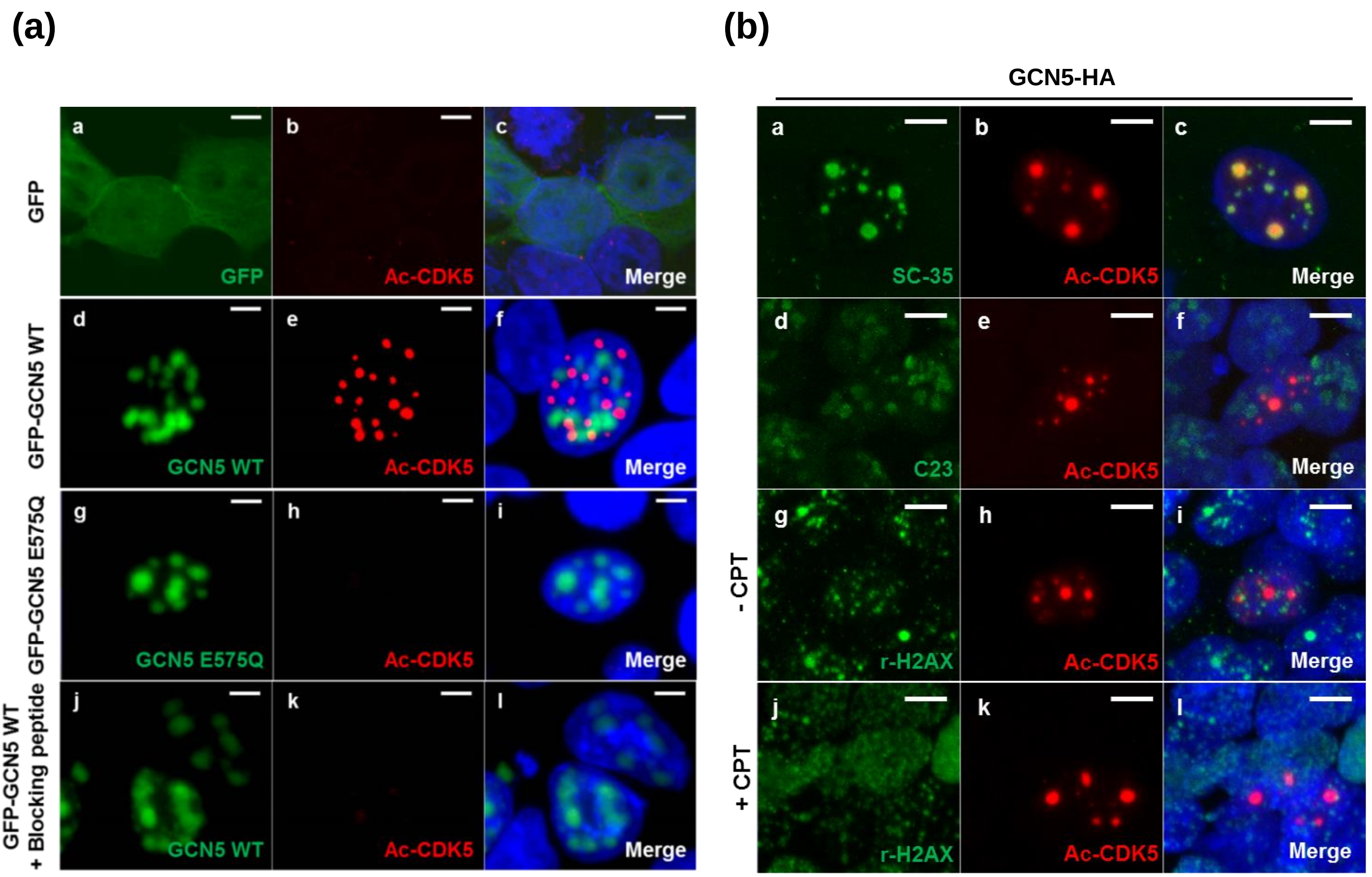

Supplementary Figure 4. Co-localization of GCN5-induced Ac-CDK5 foci with the spliceosomal component 35 (SC35). (a) HEK293 cells transfected with (a-c) GFP blank, (d-f) GFP-GCN5 WT or (g-i) a catalytically inactive mutant (GCN5 E575Q) were processed for immunostaining with an anti-Ac-CDK5 antibody followed by incubation with Alexa-594-conjugated goat anti-rabbit IgG. (j-l) The anti-Ac-CDK5 antibody was blocked with EIVAL(acK)RVRLD. Nuclei were visualized with Hoechst dye. Fluorescence images were obtained with an LSM700 confocal microscope. The scale bar represents 5 μm. **(b)** HEK293 cells transfected with GCN5-HA were processed for double immunofluorescent Ac-CDK5 localization in combination with (a-c) SC-35, (d-f) the nucleolar protein C23 or (g-l) phospho-histone H2AX (Ser139, γ-H2AX). (j-l) To induce DNA damage, HEK293 cells were exposed to camptothecin (10 μM, Sigma) for 1 hr. The following primary antibodies were used: goat polyclonal anti-SC35 (E16, 1:200; Santa Cruz Biotechnology), mouse monoclonal anti-C23 (MS-3; 1:200; Santa Cruz Biotechnology), and mouse monoclonal anti-phospho-Histone H2AX (JBW301; 1:200; Millipore). The scale bar represents 5 μm.

(a)

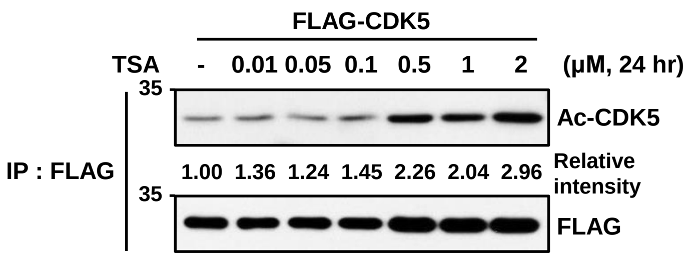

(b)

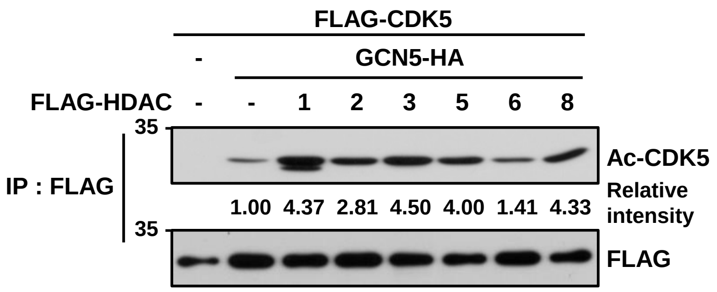

Supplementary Figure 5. Role of HDAC class I and II in Ac-CDK5 deacetylation. (a) HEK293 cells were transfected with FLAG-CDK5 and treated with trichostatin A (TSA) at the indicated doses for 24 hrs. After IP with an anti-FLAG antibody, Ac-CDK5 was measured by IB. The relative fold intensities were calculated from the ratio of Ac-CDK5 to FLAG-CDK5 over untreated controls (value=1). **(b)** HEK293 cells were co-transfected with FLAG-CDK5 and GCN5-HA plus one of the indicated FLAG-HDACs. The lysates were subjected to IP with an anti-FLAG antibody and IB with an anti-Ac-CDK5 or anti-FLAG antibody. After normalization to FLAG-CDK5, the fold intensity of Ac-CDK 5 versus control (value=1) was determined.

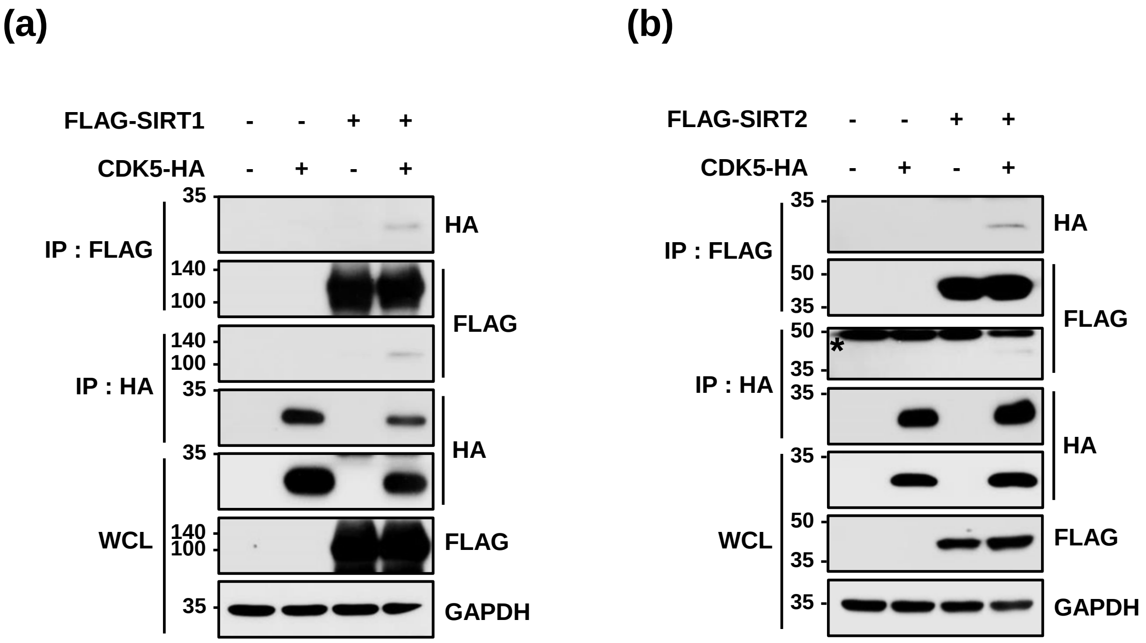

Supplementary Figure 6. Binding between CDK5 and SIRT1 or SIRT2 in HEK293 cells. HEK293 cells cultivated on P-100 dishes were transfected with the indicated combinations of CDK5-HA and **(a)** FLAG-SIRT1 or **(b)** FLAG-SIRT2. Thirty-six hours after transfection, cells were lysed in 0.5 ml of RIPA buffer containing protease inhibitor cocktail and subjected to IP with an anti-FLAG antibody. SIRT1- or SIRT2-bound CDK5 was detected by IB with an anti-HA antibody. To assess reciprocal binding, immunoprecipitates purified with an anti-HA antibody from the same lysates were subjected to IB with an anti-FLAG antibody. The expression levels of all constructs were confirmed by IB with the indicated antibodies. The asterisk indicates non-specific bands.

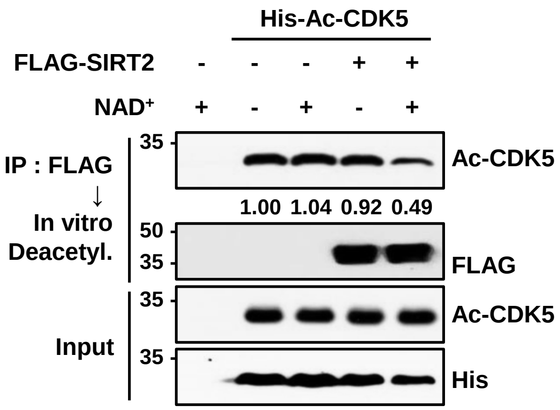

Supplementary Figure 7. Deacetylation of Ac-CDK5 by SIRT2. FLAG-SIRT2 was expressed in HEK293 cells and purified by IP with an anti-FLAG antibody. SIRT2-bound beads were incubated with recombinant Ac-CDK5 in the presence or absence of NAD^{+}. After incubation, in vitro deacetylation assay reaction mixtures were subjected to IB with an anti-Ac-CDK5 antibody. The relative intensity was calculated over the control (value=1) and is indicated at the bottom of the blot. Inputs were prepared from the same samples and subjected to IB with the indicated antibody.

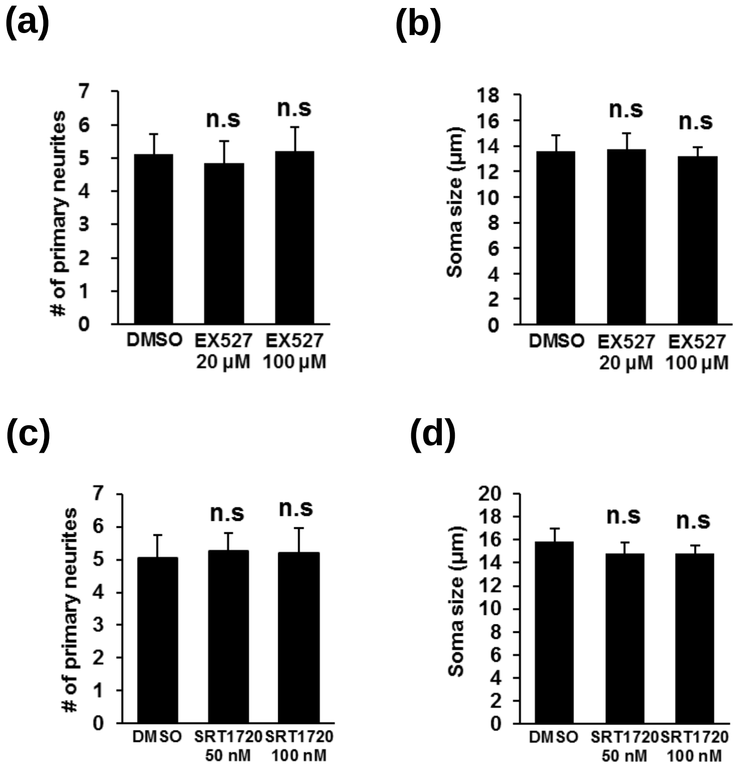

Supplementary Figure 8. Effect of EX527 or SRT1720 on primary neurite numbers and soma size. Following treatment with the indicated doses of **(a, b)** EX527 or **(c, d)** SRT1720**,** number of primary neurites and soma size were measured in hippocampal neurons cultivated as described in Figure 5. Only neurites longer than the soma size and emerging directly from the soma were considered primary neurites. Soma diameter was measured using Image-J software. The bars represent the mean±S.D. of 35-40 neurons. n.s, not significant.

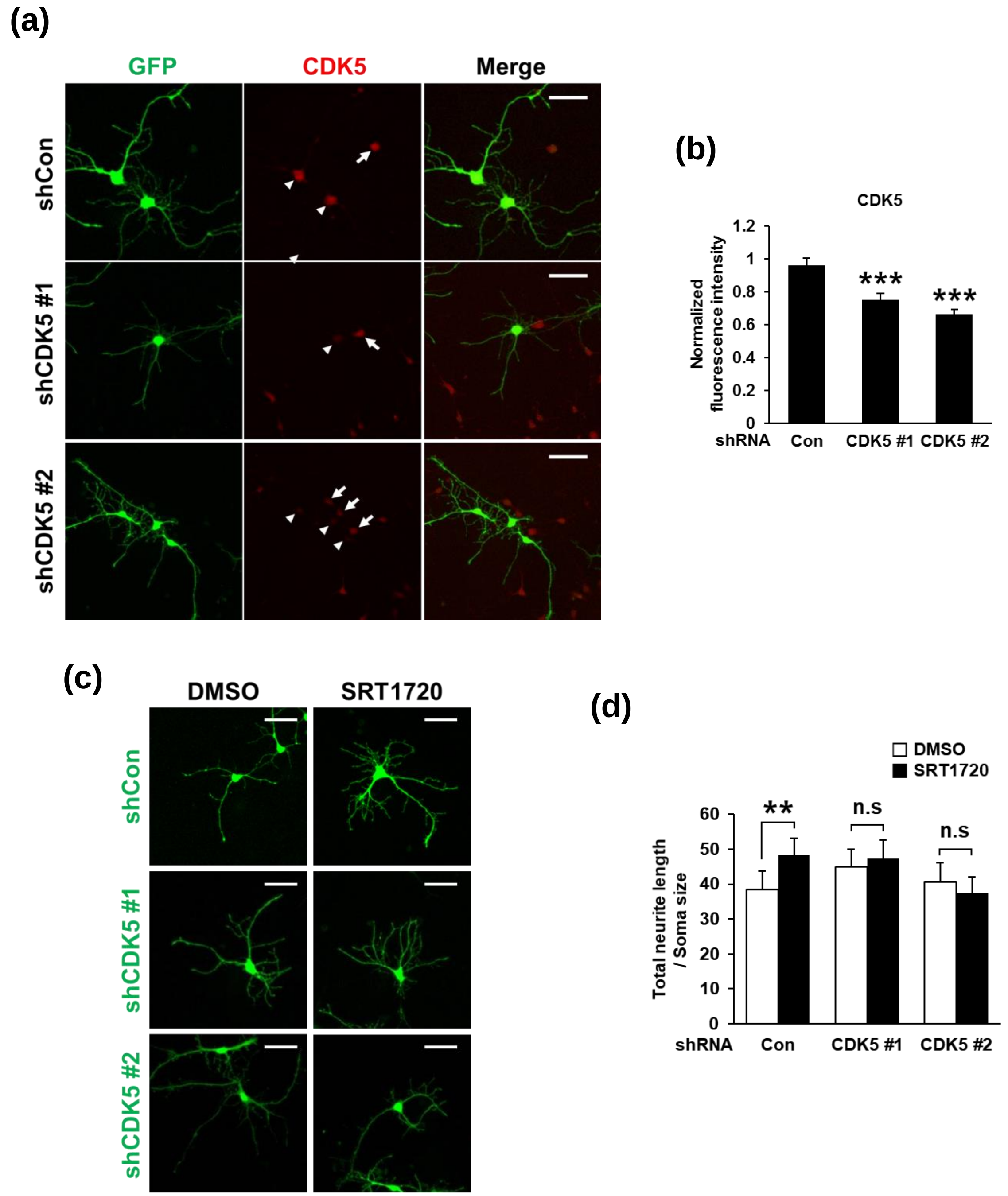

Supplementary Figure 9. CDK5 level is involved in SRT1720-mediated neurite elongation. **(a)** Hippocampal neurons transfected with GFP-expressing vector containing shRNA against CDK5 or control (scramble) were immunostained with CDK5 antibody. The GFP-expressing and non-expressing neurons are indicated by arrowheads and arrows, respectively. **(b)** To quantitate the extent of knockdown of CDK5 in shRNA-expressing neurons, fluorescent intensities of CDK5 from transfected neurons were divided with those from non-transfected neurons in each images and then expressed as the mean±S.D from at least 35-40 neurons in 10-15 randomly selected areas. ***$p<0.001$. **(c)** The shRNA-expressing neurons treated with or without 100 nM of SRT1720 for 48 hrs were fixed and observed under a confocal microscope. **(d)** Quantification of the ratio of total neurite length over soma size was performed. The bars represent the mean±S.D. from 35-40 neurons. **$p<0.01$; n.s, not significant.

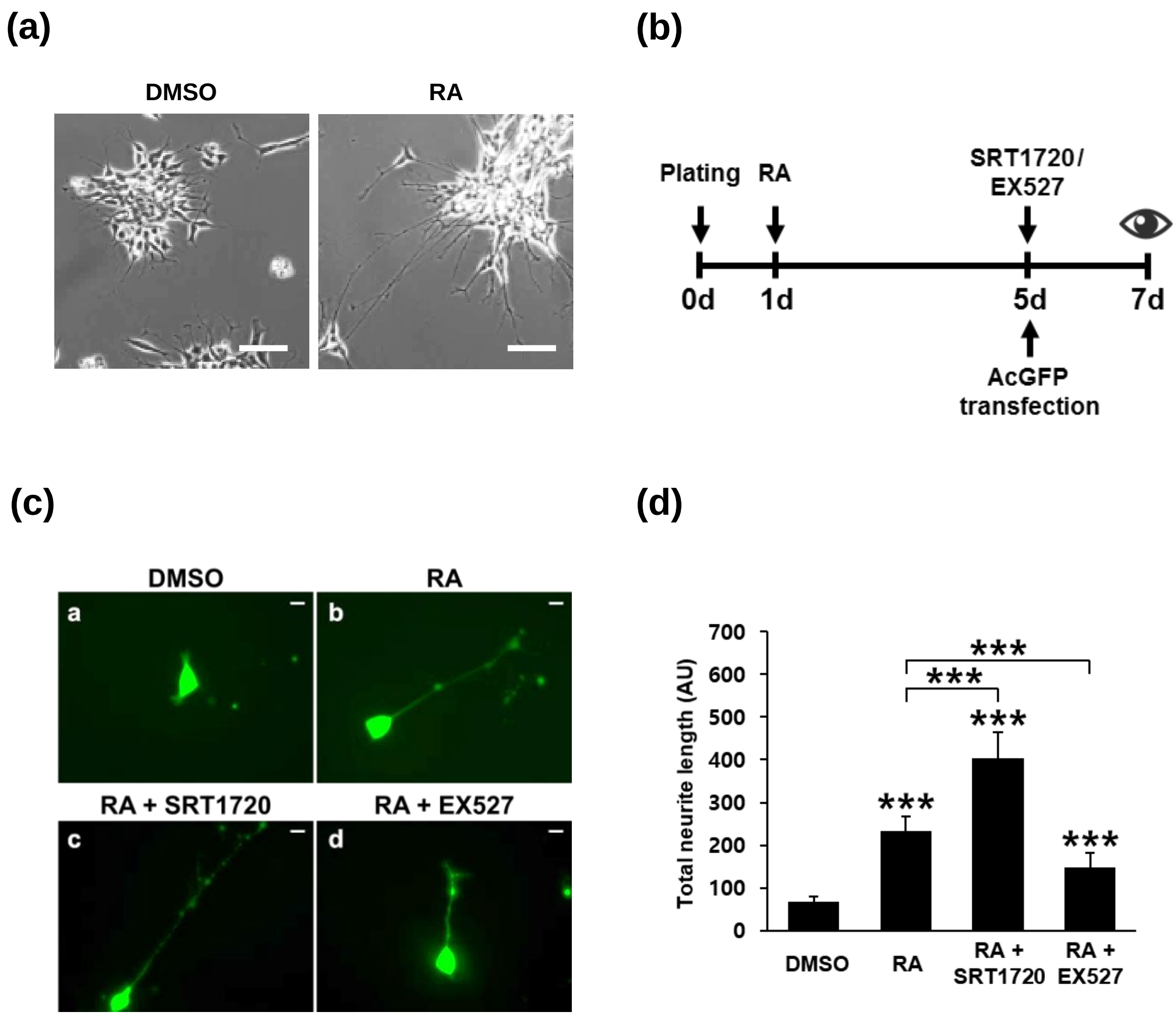

Supplementary Figure 10. Effect of EX527 or SRT1720 on total neurite length in retinoic acid-treated SH-SY5Y cells. (a) SH-SY5Y cells were maintained in DMEM supplemented with 10% heat-inactivated FBS (GenDEPOT) at 37°C in a 5% CO_2 atmosphere. To induce differentiation, cells at DIV1 were treated with 10 μM all-trans retinoic acid (RA, Sigma). Representative phase-contrast images at 6 days after RA treatment were imaged with an Axiovert 100. The scale bar represents 50 μm. **(b)** Schematic flow for the measurement of neurite elongation in RA-treated SH-SY5Y cells. As depicted, SH-SY5Y cells at DIV1 were treated with 10 μM all-trans RA for an additional 4 days and, using Lipofectamine 2000, were transfected with the pAcGFP C1 vector to visualize individual neurites. **(c)** RA-treated SH-SY5Y cells were treated for 48 hrs with 100 nM SRT1720 or 50 μM EX527. The cells were fixed with 4% paraformaldehyde for 10 min. Representative fluorescent images were obtained with an Axiovert 100. The scale bar represents 5 μm. **(d)** More than 30 cells per sample were imaged with an Axiovert 100. The total neurite lengths of individual cells were measured using Image-J software. The bars represent the mean±S.D. of 35-40 cells. $^{***}p<0.001$.

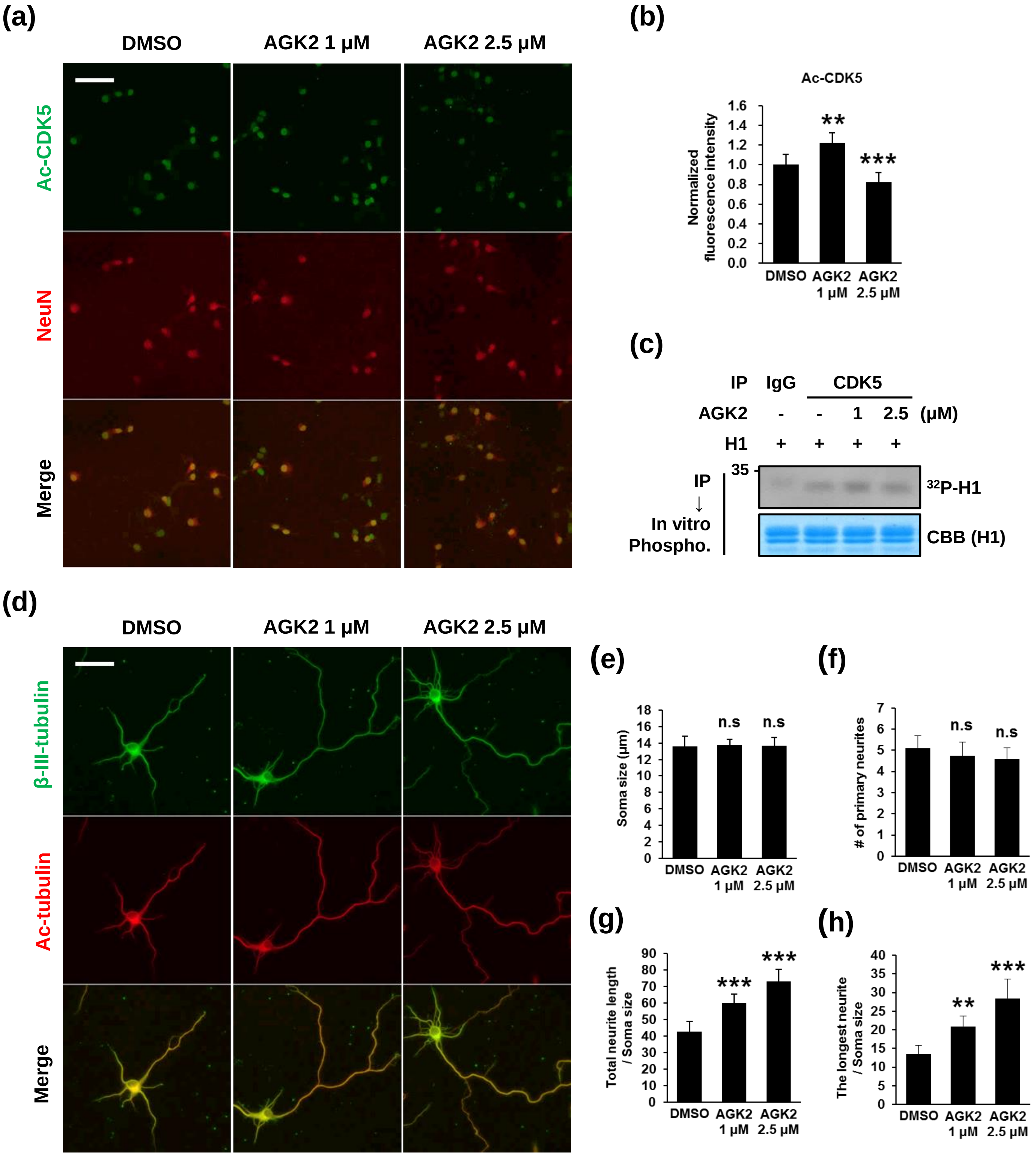
(a)
DMSO
AGK2 1 μM
AGK2 2.5 μM
Ac-CDK5
NeuN
Merge
(b)
Ac-CDK5
Normalized fluorescence intensity
1.6
1.4
1.2
1.0
0.8
0.6
0.4
0.2
0.0
**
***
DMSO
AGK2 1 μM
AGK2 2.5 μM
(c)
IP
IgG
CDK5
AGK2
-
-
1
2.5
(μM)
H1
+
+
+
+
35
IP
↓
In vitro Phospho.
32P-H1
CBB (H1)
(d)
DMSO
AGK2 1 μM
AGK2 2.5 μM
β-III-tubulin
Ac-tubulin
Merge
(e)
Soma size (μm)
n.s
n.s
DMSO
AGK2 1 μM
AGK2 2.5 μM
(f)
# of primary neurites
n.s
n.s
DMSO
AGK2 1 μM
AGK2 2.5 μM
(g)
Total neurite length / Soma size
***
***
DMSO
AGK2 1 μM
AGK2 2.5 μM
(h)
The longest neurite / Soma size
**
***
DMSO
AGK2 1 μM
AGK2 2.5 μM

Supplementary Figure 11. Inhibition of SIRT2 stimulates neurite outgrowth but does not affect CDK5 activity. (a) Rat primary hippocampal neurons at DIV3 were treated with AGK2 at the indicated concentrations for 48 hrs and immunostained with anti-Ac-CDK5 and anti-NeuN antibodies. Fluorescent images were obtained with an LSM700 confocal microscope. The scale bar represents 50 μm. **(b)** The fluorescence intensity of Ac-CDK5 in the nuclei of NeuN-positive neurons was measured using Image-J software. The relative fluorescence intensity is expressed as the fold-change over the control (value=1). The bars represent the mean±S.D of 35-40 neurons from at least 5 randomly selected areas. $^{***}p<0.001$; $^{**}p<0.01$. **(c)** Lysates obtained from hippocampal neurons treated with the indicated doses of AGK2 were subjected to IP with an anti-CDK5 antibody followed by an in vitro phosphorylation assay in the presence of H1 and [γ-^{32}P]ATP. Phosphorylated H1 levels were visualized by autoradiography. **(d)** Neurons under the same conditions as described in **(a)** were immunostained with anti-β-III-tubulin and acetylated-tubulin antibodies (T6793, 1:500, Sigma). The images obtained by confocal microscopy were analyzed using Image-J software to measure soma size and neurite outgrowth patterns. The bars represent the mean±S.D. of **(e)** the soma size, **(f)** the number of primary neurites, **(g)** total neurite length and **(h)** the length of the longest neurite normalized to soma size. The bars represent the mean±S.D of 35-40 neurons from at least 5 randomly selected areas. $^{***}p<0.001$, $^{**}p<0.01$, n.s, not significant. The scale bar represents 50 μm.

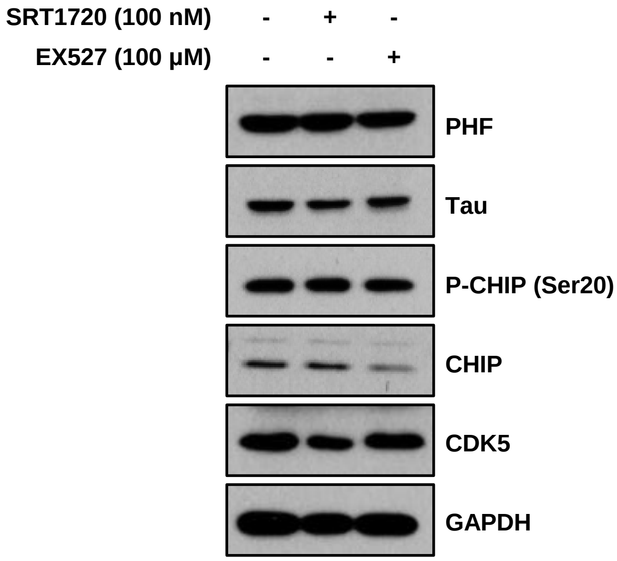

Supplementary Figure 12. Neither SRT1720 nor EX527 affect to the phosphorylation of the cytosolic CDK5 substrates in hippocampal neurons. Hippocampal neurons at DIV3 were incubated for 48 hr in the presence or absence of the indicated drugs. Cells were then subjected to IB with the indicated antibodies.

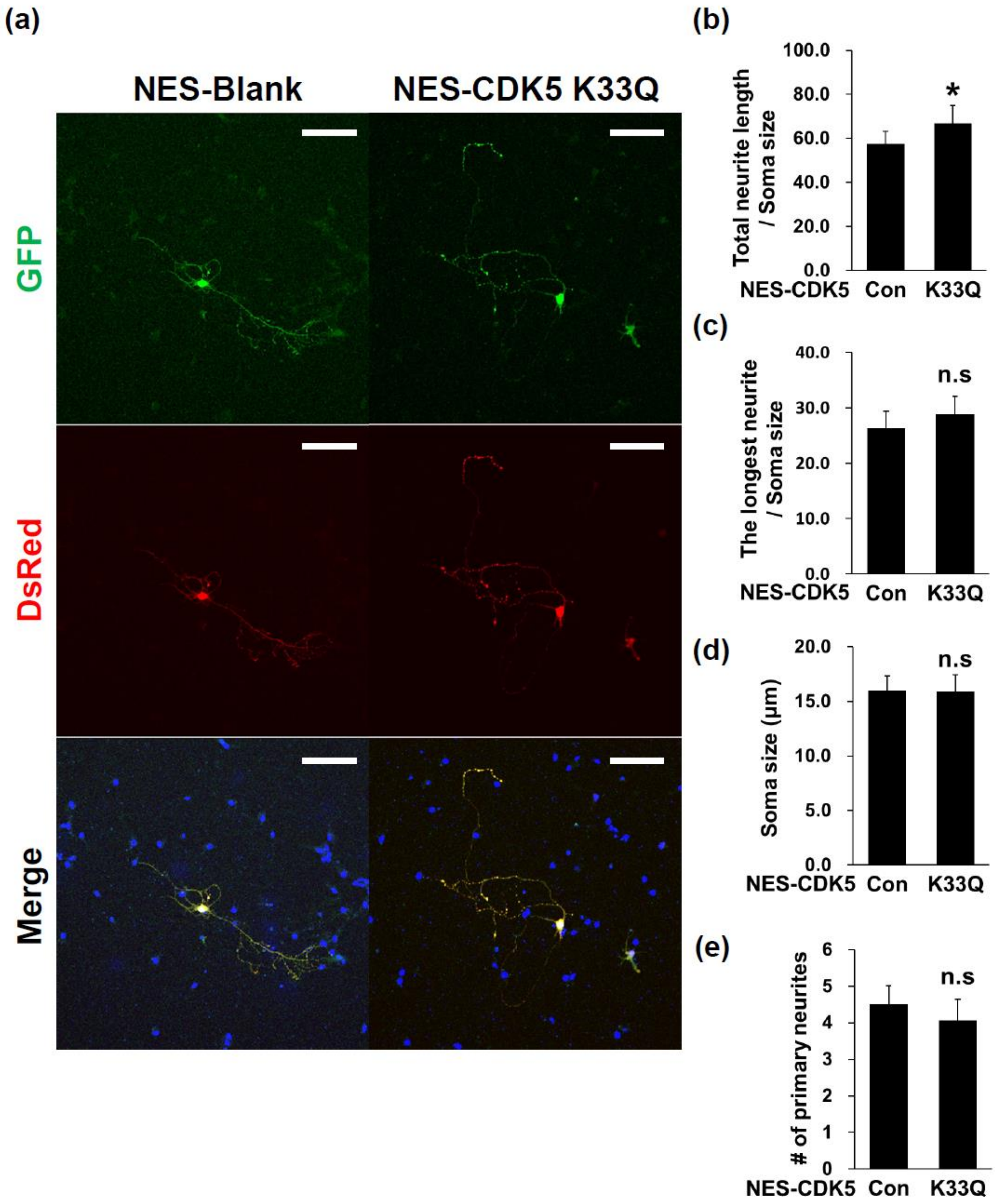

Supplementary Figure 13. Forceful localization of CDK5 K33Q into the cytosolic region fails to inhibit neurite outgrowth. (a) Hippocampal neurons at DIV3 were transfected with nuclear export signal (NES)-GFP or NES-GFP-CDK5 K33Q along with the DsRed vector. After 48 hrs, cells were fixed and examined under a confocal microscope. The scale bars represent 50 μm. Confocal images of **(b)** total neurite length, **(c)** the longest neurite length, **(d)** soma size and **(e)** the number of primary neurites were analyzed using Image-J software. The bars represent the mean±S.D. of 35-40 neurons from at least 5 randomly selected areas. $^{*}p<0.05$; n.s, not significant.

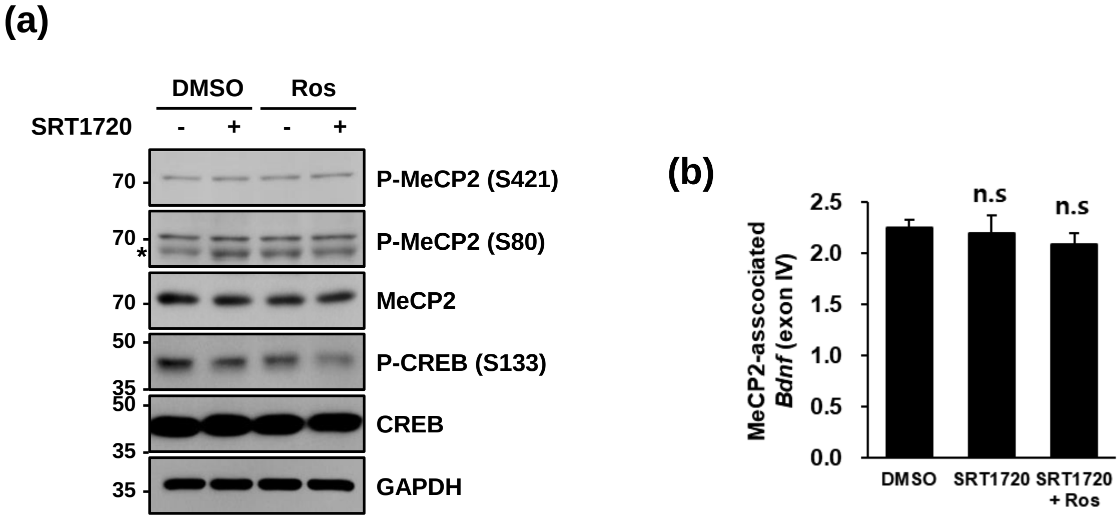

Supplementary Figure 14. SRT1720 does not affect to the phosphorylation of MeCP2 and CREB, known transcriptional regulators for *bdnf* gene. (a) Primary cultures of rat cortical neurons were treated with 100 nM SRT1720 for 24 hrs followed by adding 10 μM roscovitine (Ros) for an additional 24 hrs. Cells were lysed and subjected to IB using the indicated antibodies. **(b)** The cultures were processed for ChIP assays using primers depicted in Fig.9f, as described in the Methods section. The values are expressed as the fold change over the IgG control (value=1). The bars represent the mean±S.D. from three independent experiments. n.s., not significant

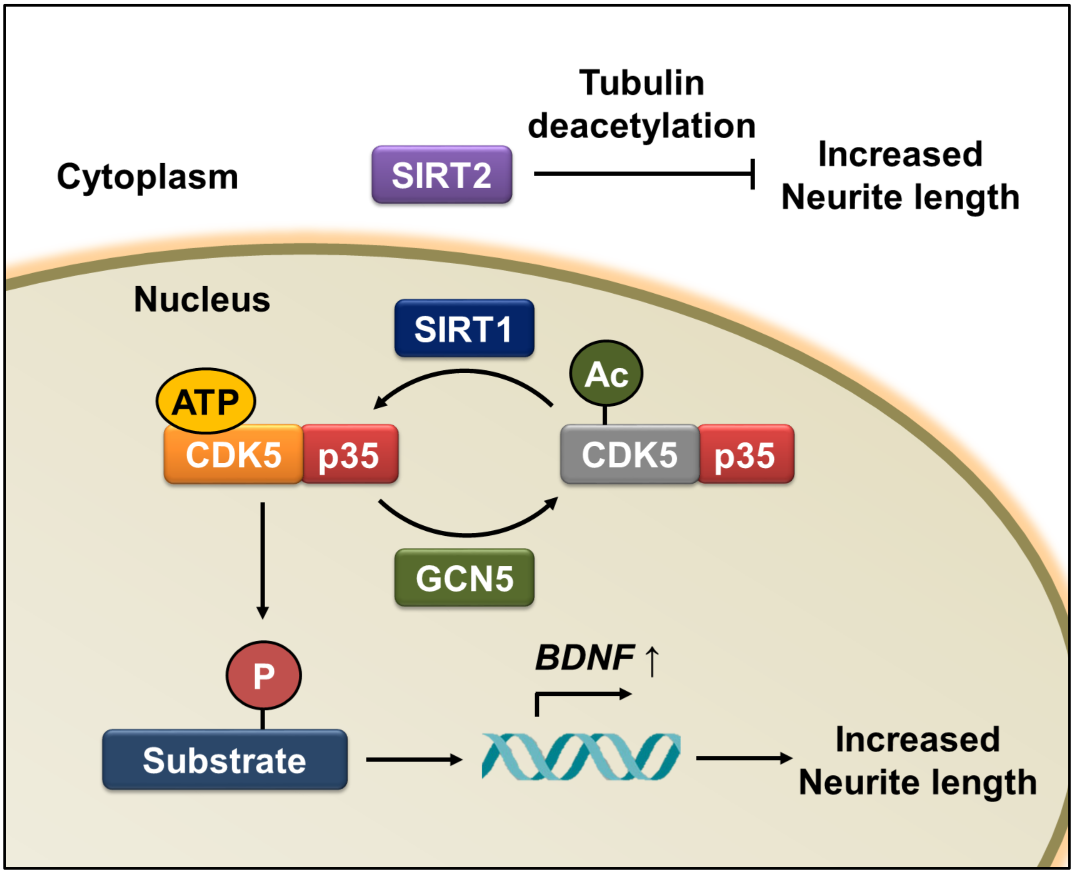

Supplementary Figure 15. A proposed model for regulation of neurite length by CDK5 (de)acetylation. Nuclear CDK5 is acetylated at K33 by GCN5. The acetylation targets ATP-binding pocket of the kinase and abolish its kinase activity. In contrast, the SIRT1-mediated deacetylation of nuclear CDK5 enhances its kinase activity. In cultured embryonic hippocampal neurons, nuclear levels of acetylated CDK5 negatively correlate with neurite length. Increased neurite length is mediated by enhanced transcriptional regulation of brain-derived neurotrophic factor (BDNF) via an unknown mechanism independent of MeCP2 and CREB phosphorylation. This event contrasts the SIRT2-mediated inhibition of neurite outgrowth via deacetylation of tubulin in the cytosol demonstrated by other groups (North et al, Mol Cell 11 (2003):437-44; Pandithage et al, J Cell Biol 180 (2008):915-29).

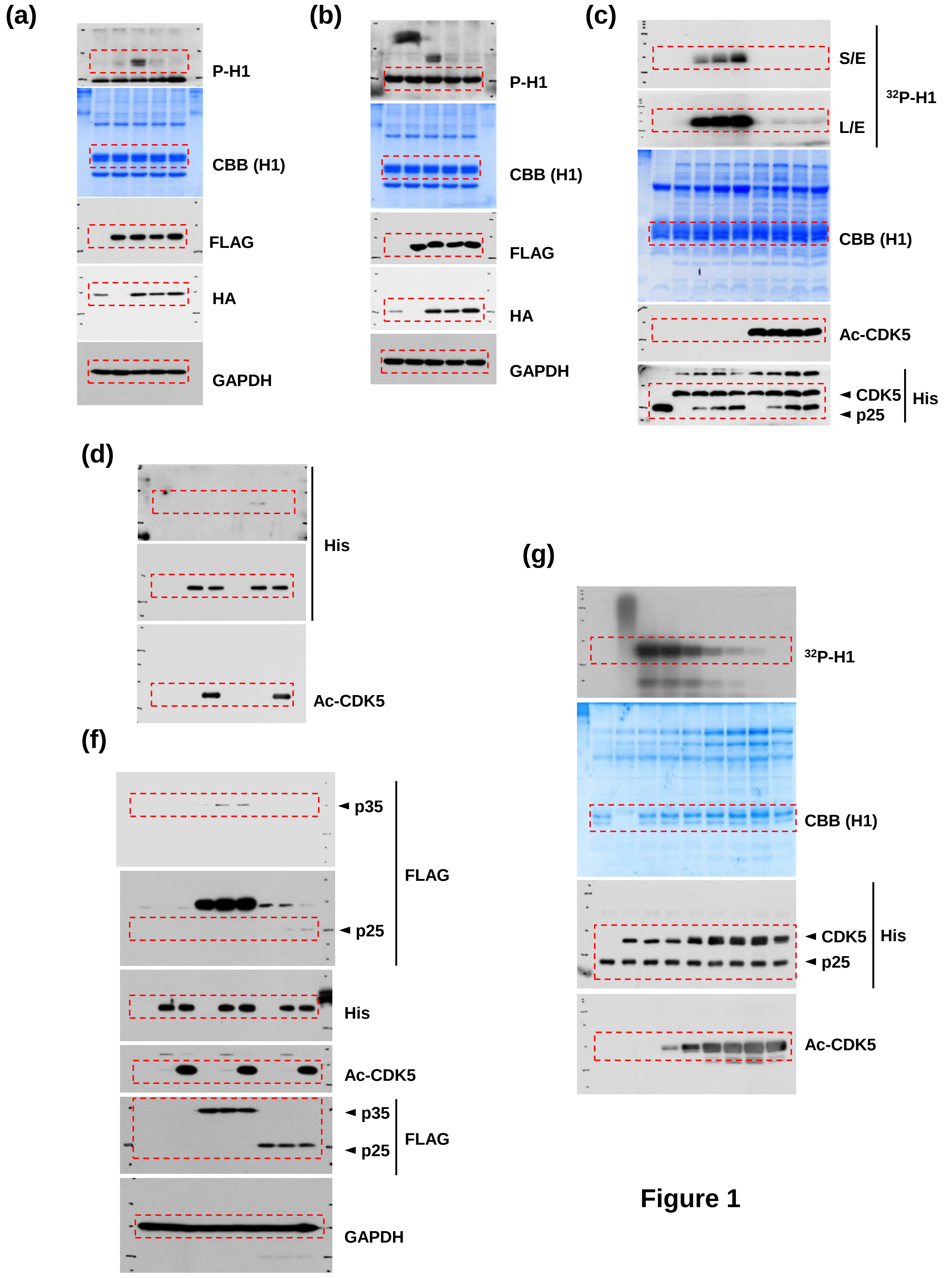
(a)
P-H1
CBB (H1)
FLAG
HA
GAPDH
(b)
P-H1
CBB (H1)
FLAG
HA
GAPDH
(c)
S/E
L/E
32P-H1
CBB (H1)
Ac-CDK5
CDK5
p25
His
(d)
His
Ac-CDK5
(f)
p35
p25
FLAG
His
Ac-CDK5
p35
p25
FLAG
GAPDH
(g)
32P-H1
CBB (H1)
CDK5
p25
His
Ac-CDK5

Figure 1

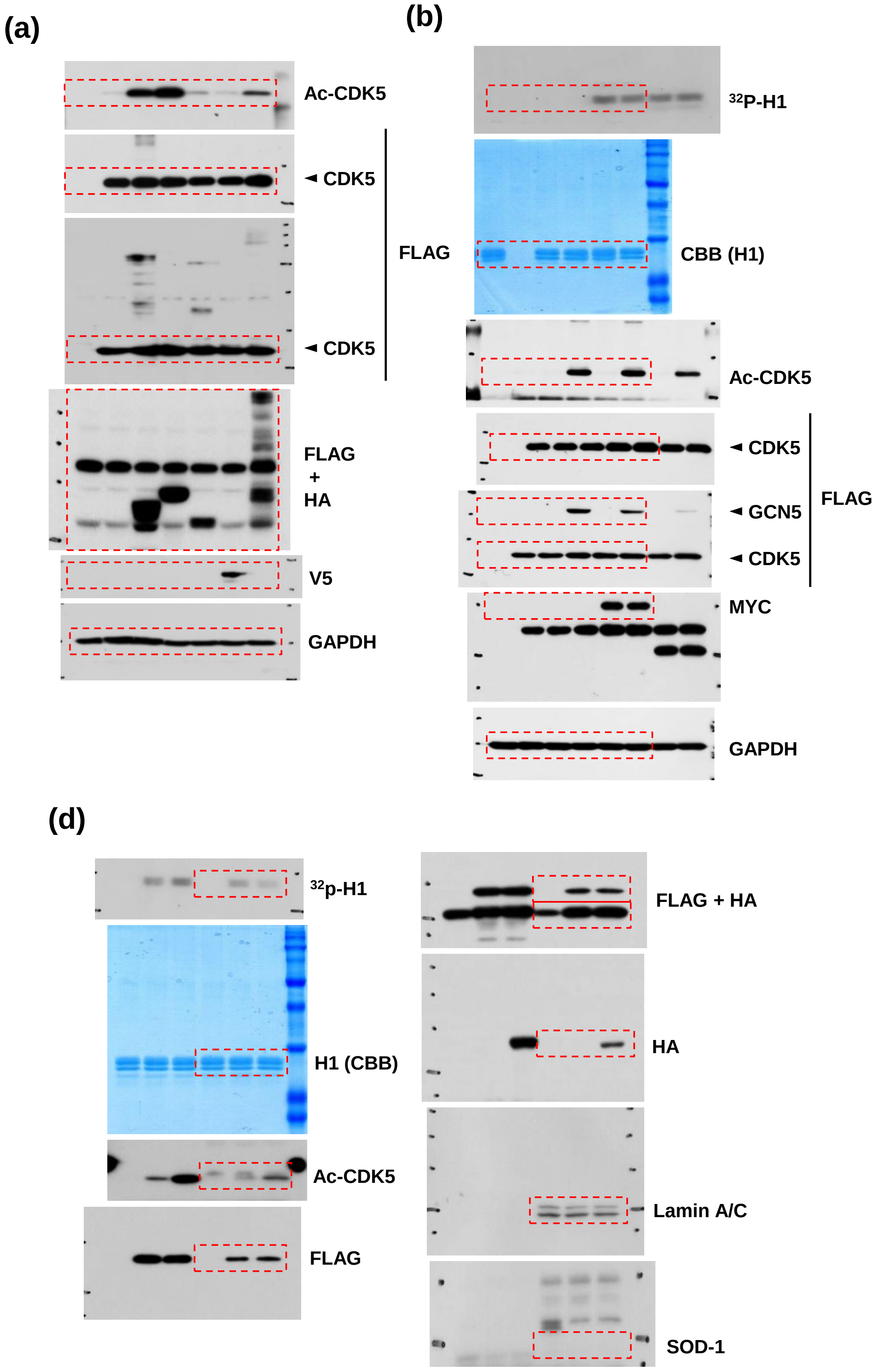
(a)
Ac-CDK5
◄ CDK5
◄ CDK5
FLAG
FLAG
+
HA
V5
GAPDH
(b)
32P-H1
CBB (H1)
Ac-CDK5
◄ CDK5
◄ GCN5
◄ CDK5
FLAG
MYC
GAPDH
(d)
32p-H1
H1 (CBB)
Ac-CDK5
FLAG
FLAG + HA
HA
Lamin A/C
SOD-1

Figure 2

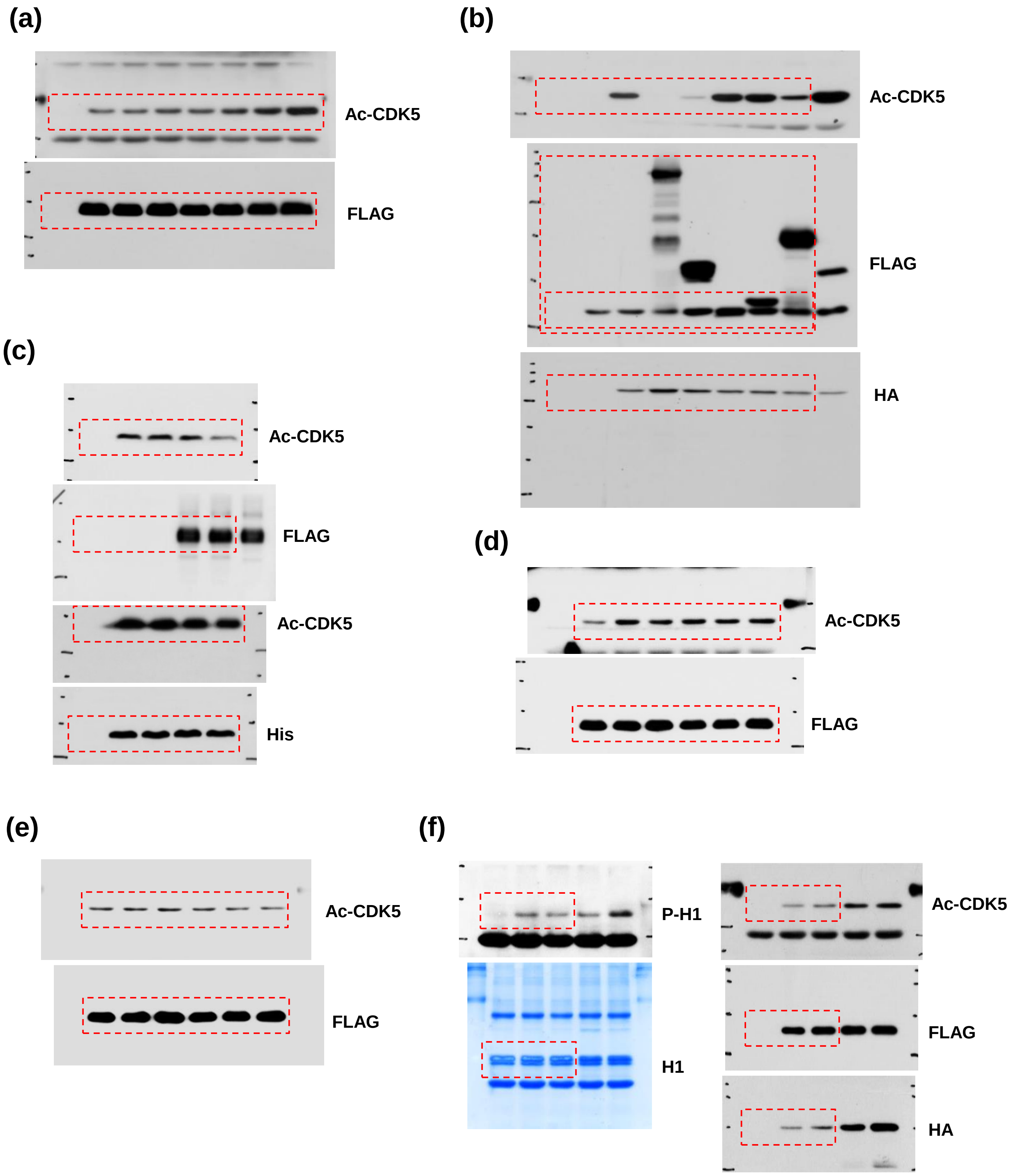
(a)
Ac-CDK5
FLAG
(b)
Ac-CDK5
FLAG
HA
(c)
Ac-CDK5
FLAG
Ac-CDK5
His
(d)
Ac-CDK5
FLAG
(e)
Ac-CDK5
FLAG
(f)
P-H1
H1
Ac-CDK5
FLAG
HA

Figure 3

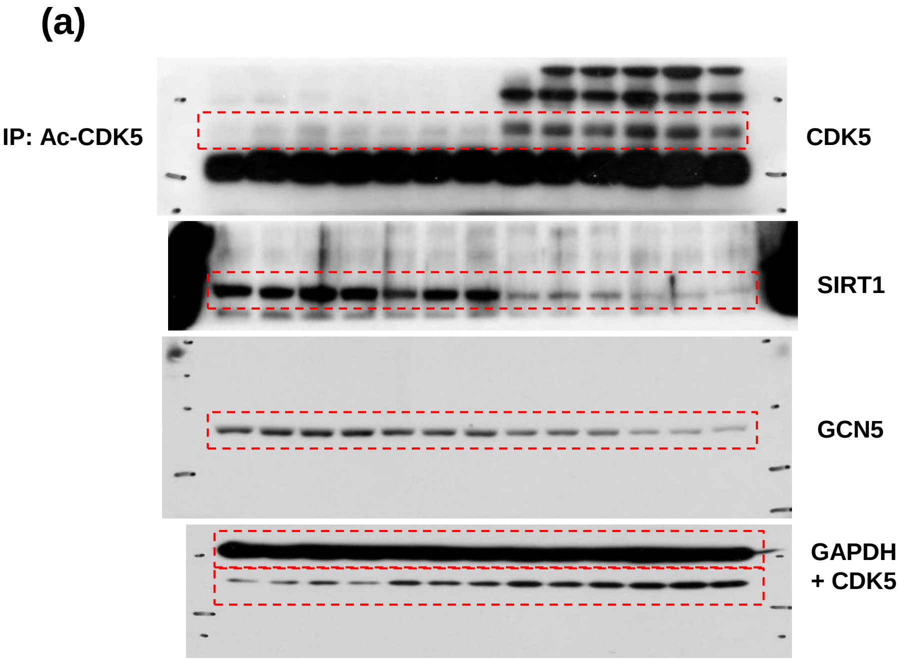

Figure 4

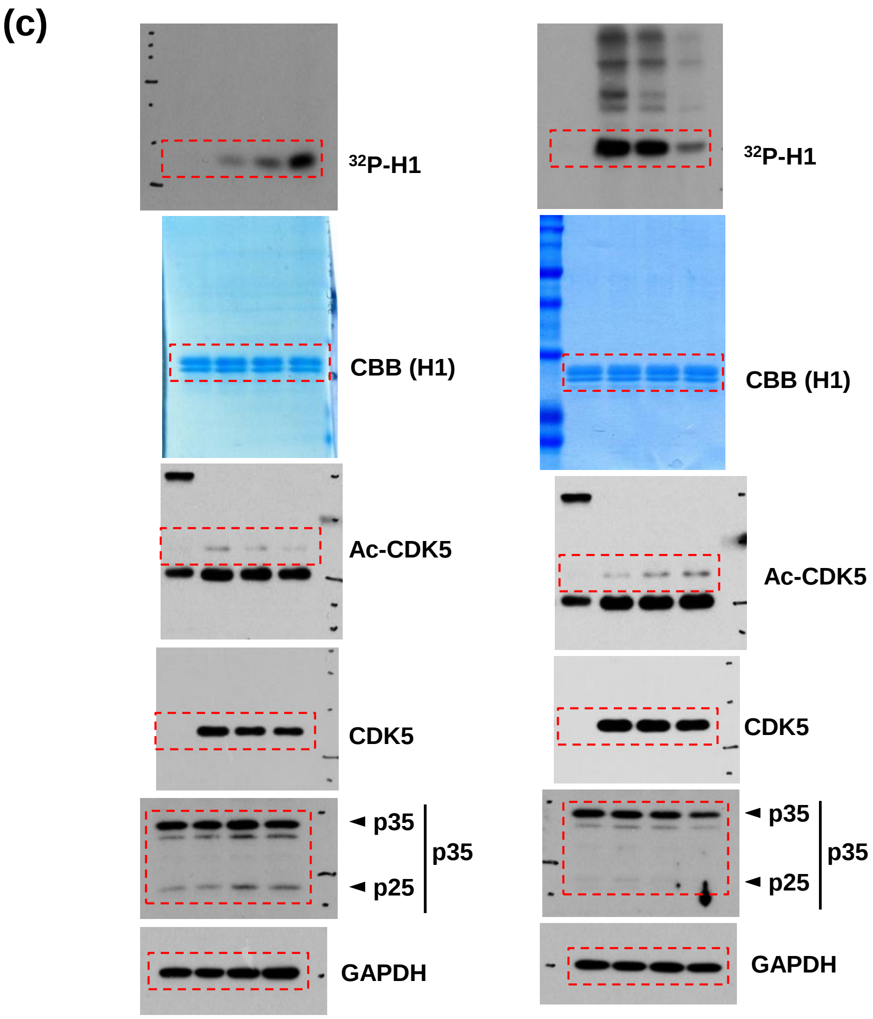

Figure 5

Suppl. Figure 2b

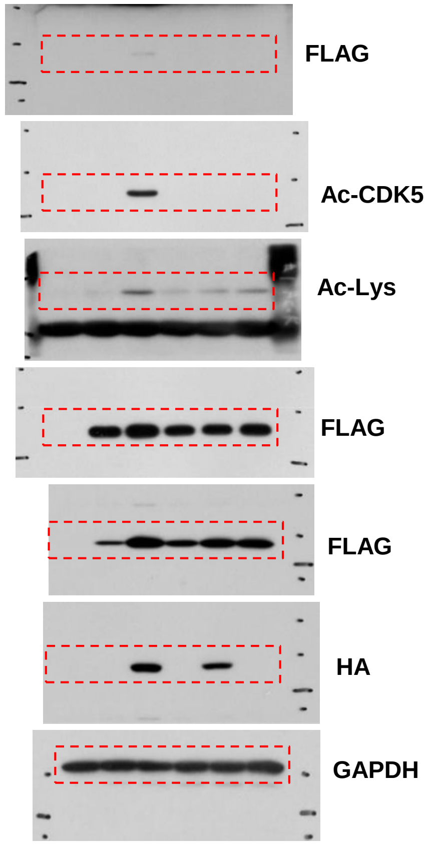

Suppl. Figure 6b

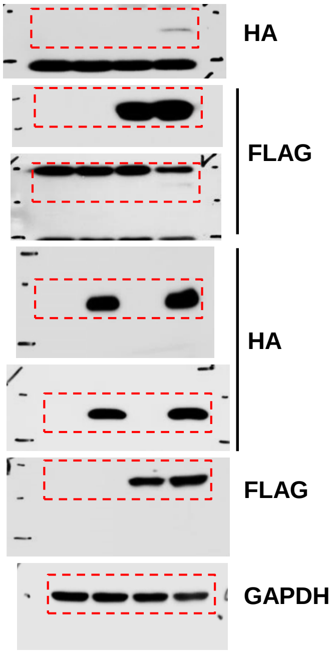

Suppl. Figure 5a

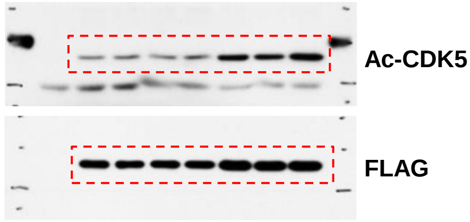

Suppl. Figure 5b

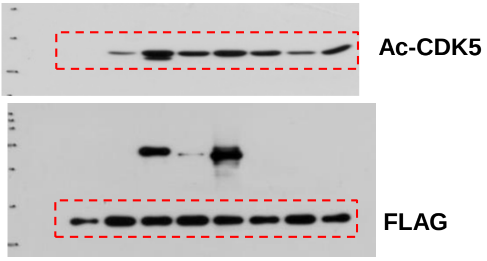

Suppl. Figure 7

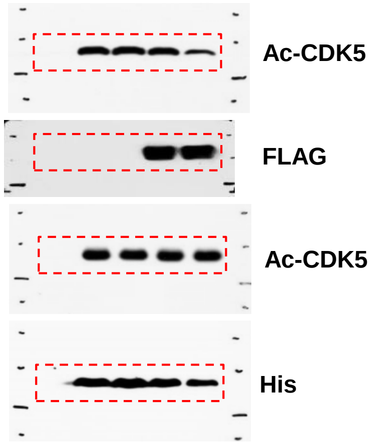

Suppl. Figure 11c

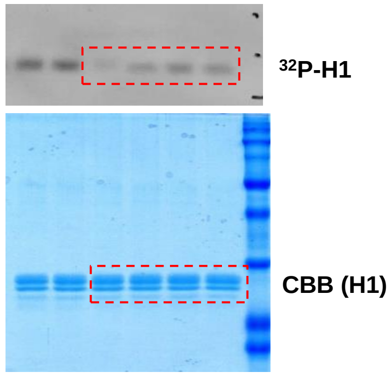

Suppl. Figure 6a

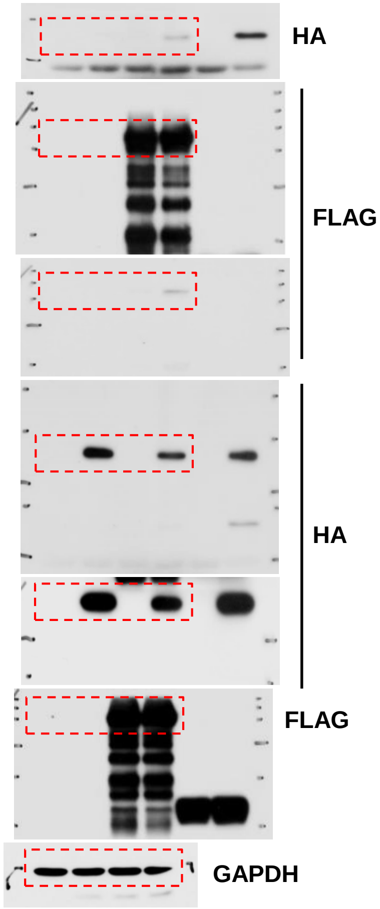

Suppl. Figure 12

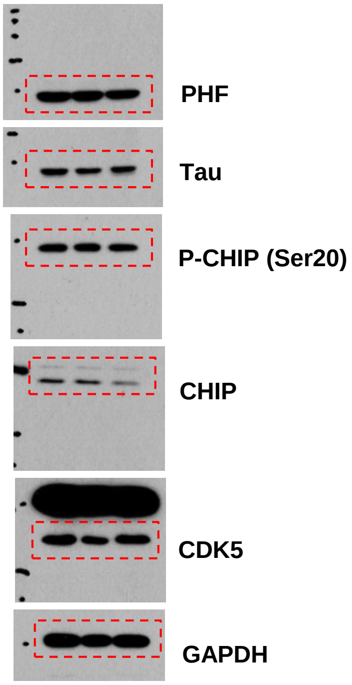

Suppl. Figure 14a

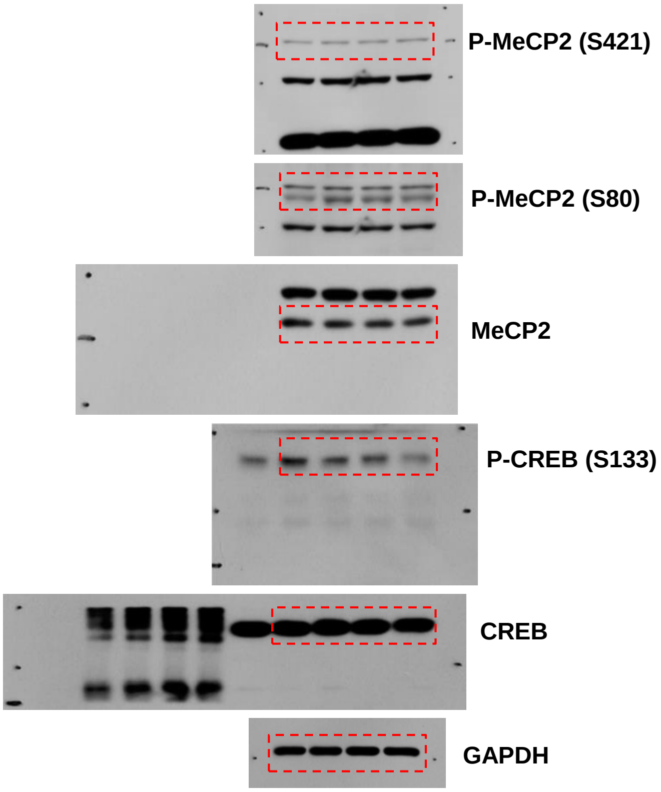

Supplement: Supplementary file 1 — Supplementary information [file 41598_2018_31785_MOESM1_ESM.pdf]
